# The global landscape of contemporary longitudinal cohorts for cardiovascular research: a systematic review

**DOI:** 10.1093/ije/dyag241

**Published:** 2026-11-16

**Authors:** Roman Roy, Janek Salatzki, Dorina-Gabriela Condurache, Marko Ristic, Adam J Lewandowski, Zahra Raisi-Estabragh

**Affiliations:** William Harvey Research Institute, National Institute for Health Research Barts Biomedical Research Centre, Queen Mary University of London, London, United Kingdom; Barts Heart Centre, St Bartholomew’s Hospital, Barts Health NHS Trust, London, United Kingdom; William Harvey Research Institute, National Institute for Health Research Barts Biomedical Research Centre, Queen Mary University of London, London, United Kingdom; Department of Cardiology, Angiology and Pneumology, Heidelberg University Hospital, Heidelberg, Germany; William Harvey Research Institute, National Institute for Health Research Barts Biomedical Research Centre, Queen Mary University of London, London, United Kingdom; Barts Heart Centre, St Bartholomew’s Hospital, Barts Health NHS Trust, London, United Kingdom; Department of Cardiology, University Clinical Centre of Serbia, Belgrade, Serbia; Nuffield Department of Population Health, Big Data Institute, University of Oxford, Oxford, United Kingdom; UK Biobank Ltd, Manchester, United Kingdom; William Harvey Research Institute, National Institute for Health Research Barts Biomedical Research Centre, Queen Mary University of London, London, United Kingdom; Barts Heart Centre, St Bartholomew’s Hospital, Barts Health NHS Trust, London, United Kingdom

**Keywords:** longitudinal studies, cohort studies, cardiovascular diseases, epidemiology, digital health, cardiovascular imaging

## Abstract

**Background:**

Longitudinal cohorts are central to cardiovascular research and increasingly integrate imaging, genomics, biomarkers, digital health, and health record linkage. This paper systematically reviews the global distribution, representativeness, and phenotypic depth of contemporary longitudinal cohorts relevant to cardiovascular research.

**Methods:**

MEDLINE, Embase (both 4 March 2025), and Global Index Medicus (20 August 2025) were systematically searched. Cohorts with cardiovascular relevance established after 2000, exclusively recruiting adults (≥18 years), with a sample size of ≥5000 participants, and ≥12 months of prospective follow-up were included. We characterized geographic distribution, diversity, phenotypic depth, and methodological features and consider implications for the future of population science.

**Results:**

From 18 185 records screened, 147 unique cohorts were identified. Of these, mean weighted age was 55 years, 54% of participants were female, and sample sizes ranged from 5000 to ∼2.5 million. Most cohorts were based in East Asia, Europe, and North America, with few from Africa and South America. Young adults and ethnicities other than White and East Asian remain under-represented. Multi-modal imaging, genetic and biomarker profiling, and digital health integration are increasingly incorporated, but remain concentrated in high-income settings. We highlight the emergence of ‘super cohorts’ with exceptional breadth and scale, cohorts with specialist cardiovascular imaging and wearable data, and multi-omics enriched resources, while examining sources of bias and challenges in harmonization.

**Conclusions:**

Contemporary cohorts offer unprecedented opportunities for cardiovascular research. Coordinated global collaboration, investment to increase representation of under-served populations, and harmonization of data standards are essential to ensure equitable, reproducible, and clinically impactful cardiovascular science.

Key Messages

- We systematically reviewed contemporary longitudinal cohorts with cardiovascular relevance to characterize their global distribution, participant representation, phenotypic depth, and methodological features.
- We identified 147 eligible cohorts established after 2000, showing significant growth in multi-modal phenotyping but also highlighting persistent heterogeneity and underrepresentation of young adults and ethnic groups other than White and East Asian.
- These findings highlight the need for global collaboration, investment to increase under-represented populations, and harmonization of data standards to ensure equitable, reproducible, and clinically impactful cardiovascular population science.

## Introduction

Longitudinal cohort studies have shaped modern understanding of cardiovascular disease (CVD), demonstrating how common exposures translate into cardiometabolic risk and future clinical events. Through repeated assessments, they establish temporal relationships, characterize disease trajectories, and inform risk prevention strategies.

In the 20th century, seminal longitudinal cohorts transformed our understanding of CVDs. The Framingham Heart Study established major modifiable risk factors, such as hypertension and high cholesterol [1]. The Whitehall Studies demonstrated the social gradient and determinants of CVD [2]. The Seven Countries Study linked international differences in cardiovascular risk to diet and environmental factors [3].

These landmark cohorts predated contemporary deep phenotyping and largely relied on periodic in-person follow-up assessments. Over the past two decades, technological advances and improvements in electronic health data infrastructures have enabled integration of traditional epidemiological data with multi-modal imaging and molecular profiling. In parallel, linkages to routine healthcare and administrative datasets allow large-scale capture of diagnoses, procedures, prescriptions, and outcomes, while digital health technologies increasingly support real-world assessment of physiology and behaviour.

Furthermore, the geographical coverage of cohorts has also broadened. While many influential cohorts from the 20th century originated in North America and Europe, longitudinal studies have increasingly emerged across low- and middle-income countries. This expansion creates opportunities to diversify the global evidence base, improve generalizability, and reduce evidence gaps. Realizing this potential requires improved visibility and systematic characterization of contemporary cohorts.

This systematic review maps the global landscape of longitudinal cohorts with cardiovascular relevance established since 2000. We characterize their geographic distribution, participant composition, and availability of contemporary data modalities, including cardiovascular imaging, omics, genetics, digital health, and electronic health record linkage. This framework provides a practical resource to support cohort discovery and highlights trends and limitations of contemporary cardiovascular population research.

## Methods

### Search strategy

Methods and results are presented according to the PRISMA 2020 statement for reporting systematic reviews [4] (Supplementary Table S1). The protocol was retrospectively registered with PROSPERO (1290508). We searched MEDLINE (via PubMed), Embase (both 4 March 2025) and Global Index Medicus (GIM, 20 August 2025) [5] to identify longitudinal cohort studies with cardiovascular relevance established after 1 January 2000. GIM enabled improved visibility of under-represented regions. Additional cohorts were identified by cross-referencing bibliographies. The full search strategy is available in the Supplementary Methods.

### Inclusion and exclusion criteria

Eligible cohorts met the following criteria: (i) exclusive recruitment of adults aged ≥18 years; (ii) start of recruitment from the year 2000 onwards; (iii) minimum of 5000 participants recruited in the first wave; (iv) prospective longitudinal cohort design, defined as at least one planned or completed follow-up assessment ≥12 months after recruitment; and (v) cardiovascular focus or relevance, defined as collection of cardiovascular exposures and/or outcomes.

We excluded: (i) retrospective, cross-sectional, and interventional studies; (ii) *post-hoc* analyses of trial populations not designed as prospective cohorts; (iii) registry- or insurance claims-only sources without study-specific consent, assessments, or follow-up; and (iv) studies with insufficient methodological detail to determine eligibility.

### Cohort selection and data extraction

All records identified through database searches were collated, and duplicates were removed. Abstracts were independently screened by four reviewers (D.-G.C., M.R., R.R., and J.S.) against the eligibility criteria. Each cohort underwent full review by one reviewer and verification by a second reviewer. Discrepancies were resolved by discussion and consensus, with arbitration by senior investigators (Z.R-E. and A.J.L.).

Data on cohort characteristics, participant demographics, and available data domains were extracted using a predesigned, standardized data extraction template (Supplementary Table S2). Recruitment waves were defined as enrolment of study-naïve participants into the same cohort. For cardiovascular imaging, we recorded availability (full cohort or subset), modality, and availability of repeat imaging. Follow-up characteristics included type (active, passive, or mixed) and mode (in-person, remote assessment, or health record linkage). Full methodological details are provided in the Supplementary Methods.

## Results

### Descriptive overview

A total of 18 185 abstracts were identified across PubMed, EMBASE, GIM, and cross-referencing (Fig. 1). After deduplication, 16 648 abstracts were screened, yielding 2075 studies with potentially eligible cohorts. Of these, 275 unique cohorts were identified for in-depth data extraction, with 147 of these meeting eligibility criteria. The commonest reasons for exclusion were sample size <5000 participants (57%), establishment before 2000 (17%), and lack of longitudinal design (10%). Cohort-level details are available in the online searchable database (Supplementary Table S3). A summary of the systematic review methodology and key findings are summarized in Fig. 2.

**Figure 1:**
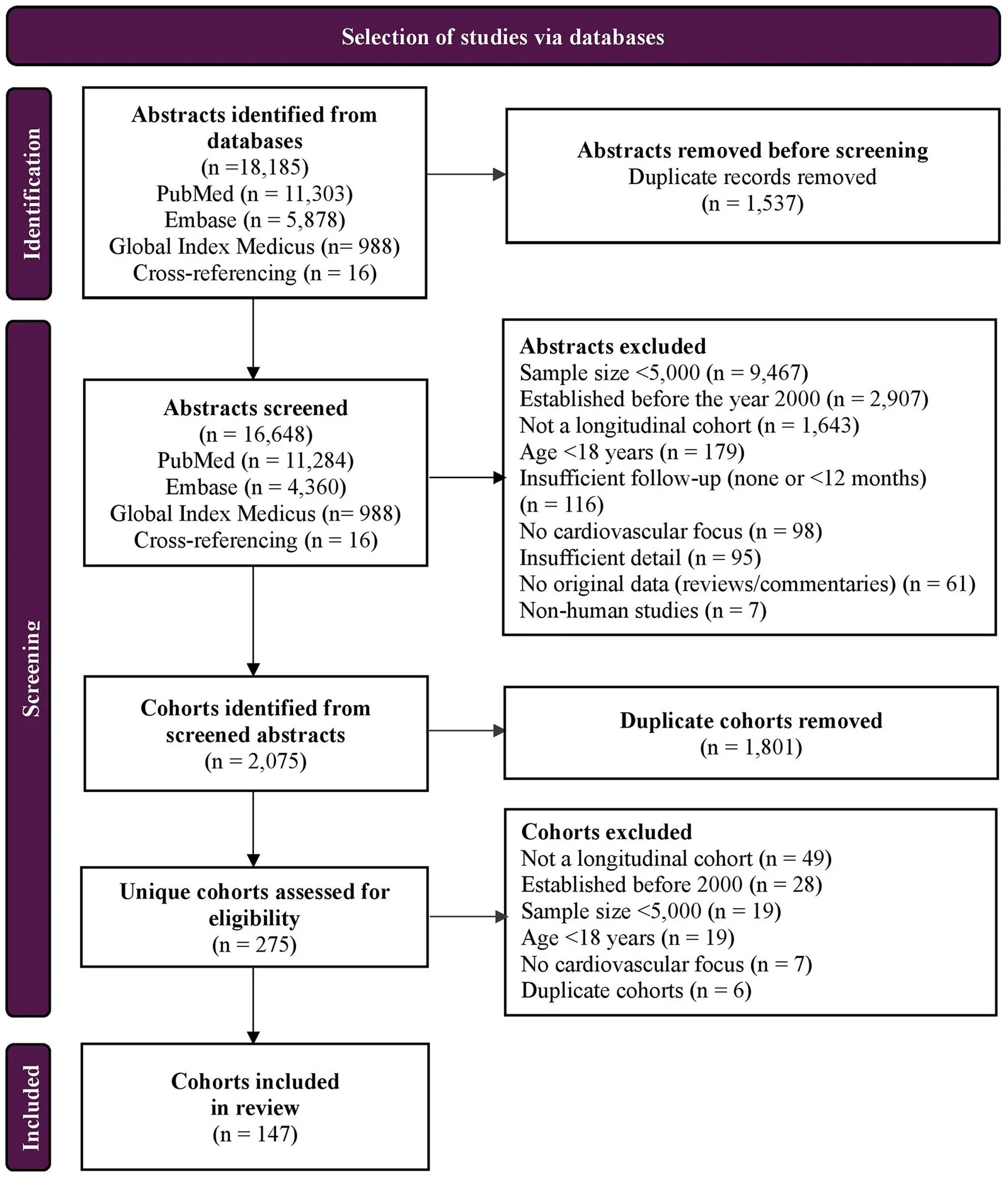
PRISMA flow diagram of study selection process. PRISMA flow diagram illustrating the identification, screening, eligibility assessment, and inclusion of longitudinal cohorts for cardiovascular research.

**Figure 2:**
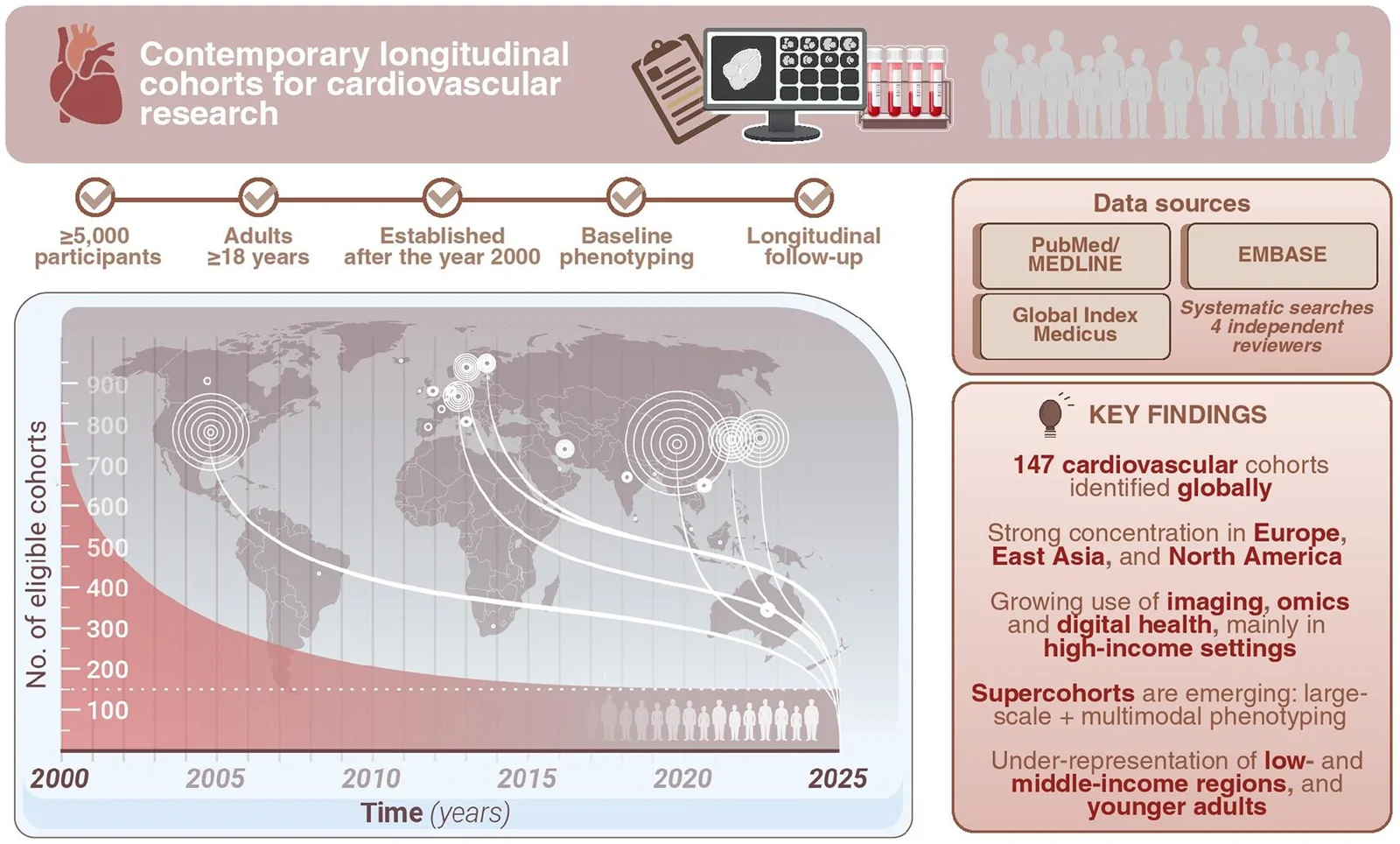
Summary of study methodology and key findings. Eligible longitudinal cohorts for cardiovascular research were identified through systematic searches of PubMed, Embase, and Global Index Medicus up to 2025. Inclusion criteria were prespecified (≥5000 adults, baseline phenotyping, longitudinal follow-up, establishment ≥2000).

#### Geographical distribution and participant selection

Most cohorts were conducted in East Asia (35%), Europe (25%), and North America (18%) (Fig. 3), with most contributions from China (*n* = 28), the USA (*n* = 20), South Korea (*n* = 9), and Japan (*n* = 9). Africa (*n* = 1) and South America (*n* = 2) were under-represented. Twelve cohorts recruited across three or more countries, predominantly from Europe, North America, and Asia. The largest cohorts were disproportionately East Asian, European, or North American, with these regions contributing 15 of 17 cohorts recruiting >200 000 participants.

**Figure 3:**
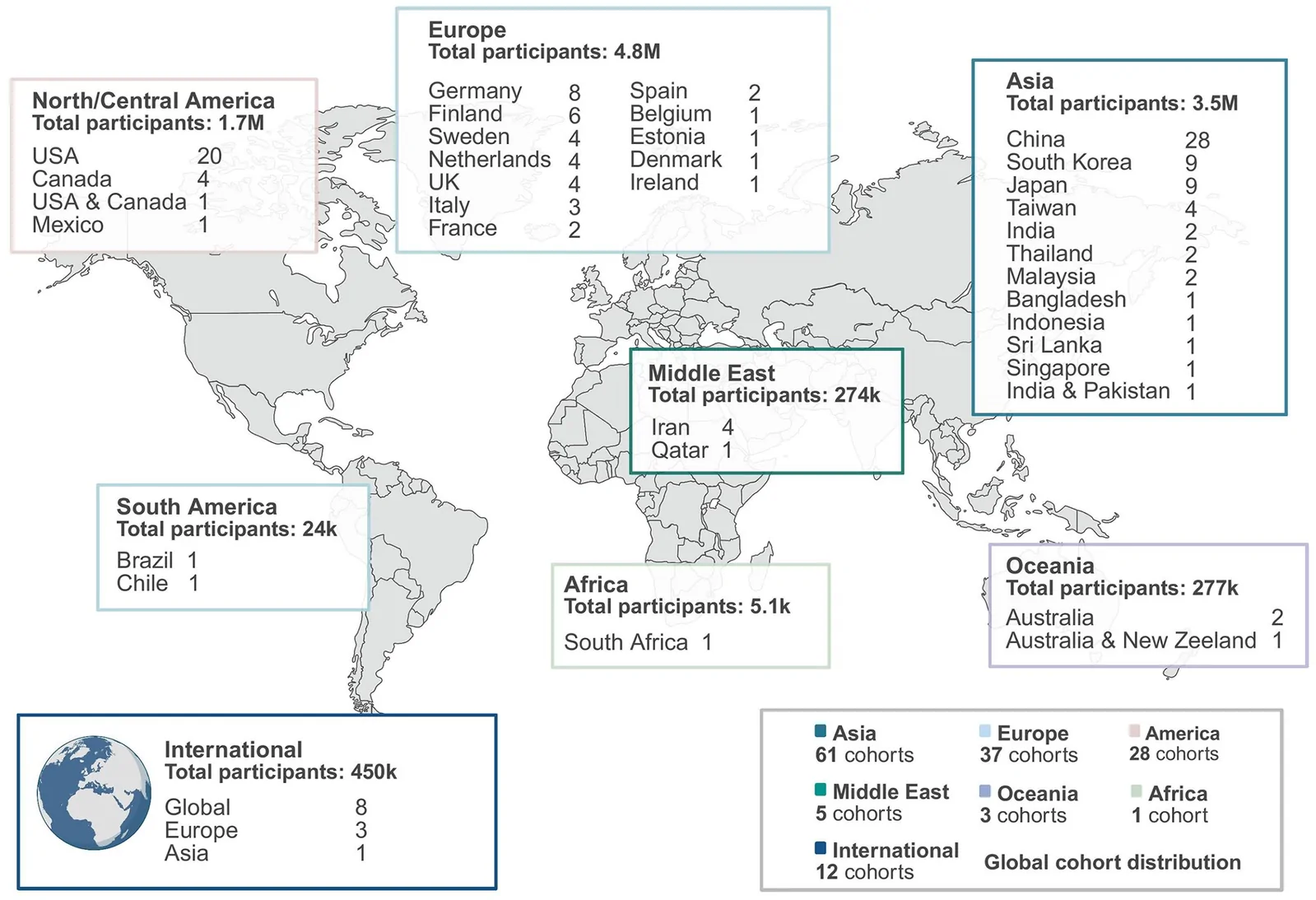
Global distribution of included longitudinal cohort studies. Global distribution of eligible longitudinal cohorts for cardiovascular research. Counts represent the number of distinct cohorts included in the review. Cohorts spanning multiple countries/continents are listed separately under ‘International’. Each cohort was counted once in the total cohort count. Total participant numbers represent the cumulative reported sample size of cohorts within each region and are rounded for display.​

Most included cohorts were population-based (67%), ranging from municipalities and regional samples to national cohorts. Clinical cohorts accounted for 38 studies (26%), encompassing case-based cohorts and disease-specific outpatient and inpatient populations. Occupational cohorts were uncommon (5%).

#### Participant characteristics

Sex distribution showed an overall modest female predominance, with the weighted average across all cohorts ∼54% female. Twenty cohorts (14%) recruited >60% males, and 24 cohorts (16%) recruited >60% females. Two studies considered males only; none were exclusively female.

Most cohorts focused on middle-aged to older populations, with mean recruitment age clustered between 50 and 70 years (Fig. 4). Thirty studies (20%) specifically investigated older populations, with mean age >65 years. Only 24 cohorts (16%) reported a mean age <50 years at recruitment, and eight cohorts (5%) < 40 years.

**Figure 4:**
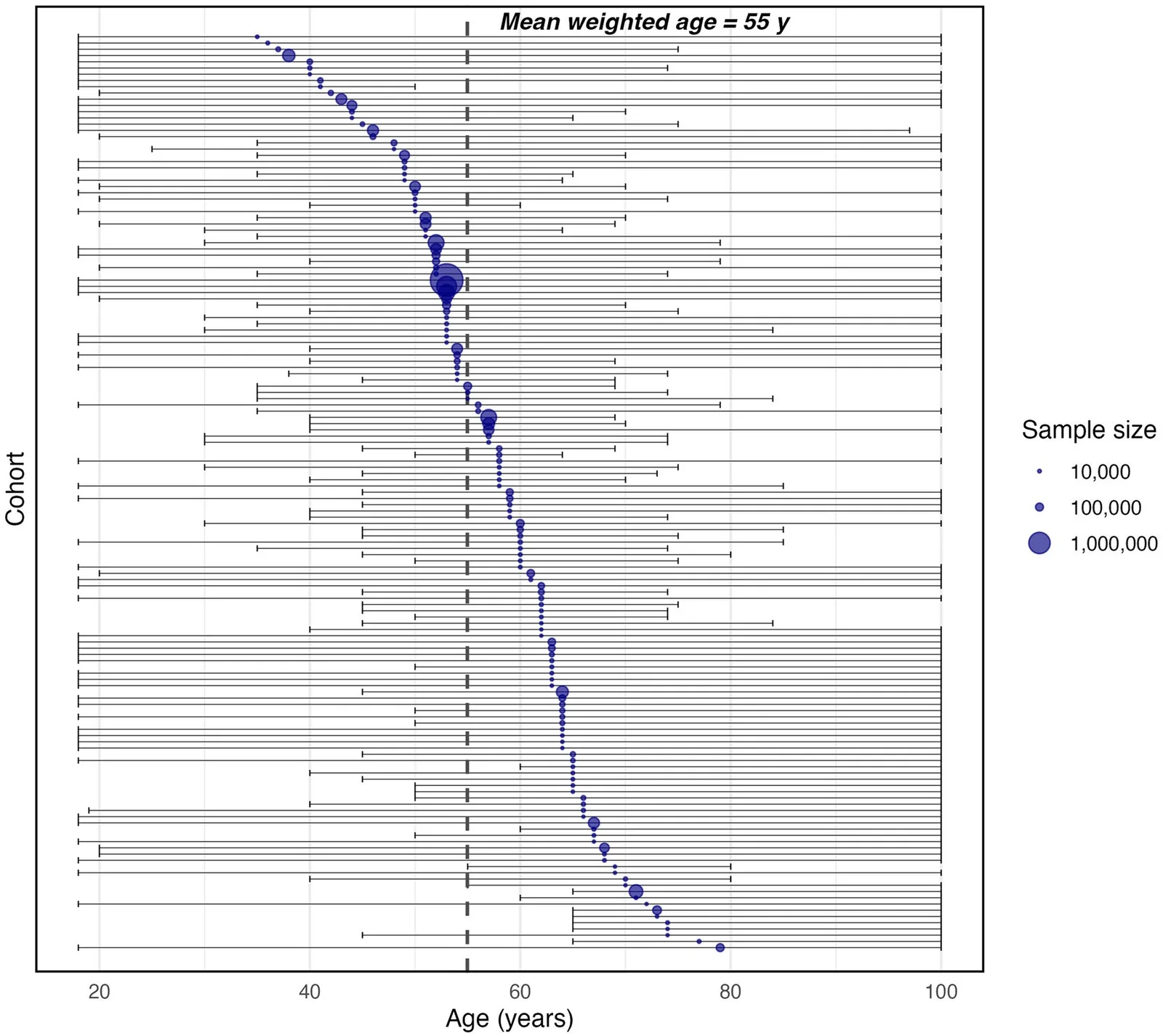
Summary of recruitment age across cohorts. Each row represents one unique cohort. Location of blue circle represents the mean age, and size of blue circle represents cohort sample size. Horizontal bars represent age range included in cohort study (i.e. inclusion criteria).​ Characteristics were identified from official cohort resources/cohort profiles, or where not available, a publication using a large, representative sample.

Ethnic composition largely reflected regional recruitment and population structures and was often homogeneous. Many East Asian cohorts largely recruited Han Chinese, Japanese, or South Korean participants, with many European studies predominantly composed of White or European ancestry participants. Explicitly multiethnic cohorts were less common and were mainly concentrated in North America, selected urban settings, and a few international cohorts.

#### Cohort size

Cohort size ranged from 5000 to almost 2.5 million participants, with five studies enrolling more than 500 000 individuals. Seventy-four cohorts (50%) enrolled 10 000–100 000 participants, and 46 (31%) enrolled 5000–10 000 participants. The largest cohorts were predominantly population-based studies linked to national health or administrative registries as part of their follow-up infrastructure.

### Recruitment timing, strategies, and waves

Recruitment started in the 2000s for 83 cohorts (56%), in the 2010s for 61 (41%), and in the 2020s for three (2%). Nineteen cohorts (13%) reported ongoing or open-ended recruitment.

Recruitment strategies varied widely, reflecting different healthcare systems and study objectives. Approaches included population-based sampling frameworks, primary care or healthcare registers, disease-specific registries, workplace-based enrolment, and mixed community-healthcare models. Population registers were frequently used to identify eligible participants and achieve representative sampling or recruit specific subgroups.

Most cohorts used a single recruitment wave followed by longitudinal follow-up (82%). Multiple recruitment waves were used in 25 cohorts (17%), often to replenish sample size, maintain population representation, or extend recruitment to additional groups. Among multi-wave cohorts, methodological differences between waves were common, including changes in recruitment strategy, eligibility criteria, assessment protocols, and follow-up mechanisms.

Follow-up approaches were heterogeneous, with active (33%), passive (8%), and predominantly mixed designs (58%), reflecting varied study design and healthcare infrastructure. Duration, frequency, and outcome ascertainment varied widely, contributing to substantial between-cohort heterogeneity. Outcome ascertainment methods were similarly heterogeneous, ranging from participant self-report [6] to confirmation using medical records or treating physicians [7], structured physician adjudication using pre-specified criteria [8], and formal endpoint adjudication committees [9]. Other approaches included ascertainment from routinely coded primary care, hospital and mortality records, and disease-specific or national registry linkage [7, 10, 11]. In some cohorts, different ascertainment approaches were used for different endpoints [7].

### Phenotyping strategies

#### Super cohorts

Fourteen cohorts formed a distinct group of large-scale, deeply phenotyped resources, which we describe here as ‘super cohorts’ (Table 1). These cohorts included dedicated cardiovascular imaging and either biomarker profiling or genetic analyses alongside detailed traditional epidemiological data for at least 25 000 participants. In many cases, these cohorts included linkage to national records for near-complete outcome ascertainment, multi-organ imaging, repeat examinations, and wearable device data. Of these, six were set in Asia [12–17], five in Europe [7, 9–11, 18], two in North America [19, 20], and one in the Middle East [21]. Several smaller but highly informative cohorts also offered rich multi-modal phenotyping [8, 22–25].

**Table 1:** Super cohorts with multi-domain phenotyping.^a^

Cohort name	Country	Sample size	Recruitment years	Ethnicity	Age range	Biosamples	Omics available	Follow-up
UK Biobank	UK	503 317	2006–2010	White (95%)	40–69 year (mean 57)	Blood, urine, saliva	Metabolomics, proteomics, genomics, epigenomics	EHRs; in person; remote assessment
Kangbuk Samsung Health Study (KSHS)	South Korea	290 000	2002–ongoing	Unspecified	≥18 years (mean 38)	Blood, urine, stool	Genomics	EHRs; in person
Ontario Health Study (OHS)	Canada	225 620	2009–2017	White (82%)	18–97 year (mean 46)	Blood, urine	Metabolomics, genomics, transcriptomics, proteomics	EHRs; remote assessment
Korean Genome and Epidemiology Study (KoGES)	South Korea	211 705	2001–2011	Unspecified	≥40 year (mean 52)	Blood, urine	Metabolomics, genomics, epigenomics	EHRs; remote assessment
German National Cohort (NAKO)	Germany	205 415	2014–2019	Unspecified	20–69 years (mean 51)	Blood, urine, stool, saliva, nasal swabs	Metabolomics, proteomics, genomics, serolomics	EHRs; in person; remote assessment
Copenhagen General Population Study (CGPS)	Denmark	170 000	2003–ongoing	White (100%)	20–100 years (mean 53)	Blood	Metabolomics, genomics	EHRs
Tohoku Medical Megabank Community Cohort Study	Japan	87 865	2013–2015	Unspecified	≥20 years (mean 61)	Blood, urine	Metabolomics, genomics, epigenomics	EHRs; in person; remote assessment
China National Stroke Registry (CNSR)	China	62 066	2007–2008	Chinese (97%)	>18 years (mean 64)	Blood, urine	Genomics	EHRs; in person; remote assessment
Canadian Longitudinal Study on Aging (CLSA)	Canada	51 338	2010–2015	White (92%)	45–85 years (mean 60)	Blood, urine, stool	Metabolomics, genomics, epigenomics	EHRs; in person
Hamburg City Health Study (HCHS)	Germany	45 000	2016–2022	Caucasian	45–74 years (mean 62)	Blood, urine, saliva, skin, other	Transcriptomics, proteomics, genomics, lipidomics	EHRs; in person; remote assessment
Qatar Biobank	Qatar	37 000	2013–ongoing	Qatari (76%)	≥18 years (mean 40)	Blood, urine, saliva	Metabolomics, proteomics, genomics, epigenomics, transcriptomics, virome	EHRs; in person
Centre for Cardiometabolic Risk Reduction in South Asia (CARRS)	India, Pakistan	30 847	2010–2012	Unspecified	≥20 years (mean 42)	Blood, urine, saliva	Metabolomics, planned genomics, epigenomics	EHRs; in person; remote assessment
Guangzhou Biobank Cohort Study (GBCS)	China	30 430	2003–2008	Unspecified	>50 years (mean 64)	Blood, urine	Genomics	EHRs; in person; remote assessment
Swedish Cardiopulmonary BioImage Study (SCAPIS)	Sweden	30 154	2013–2018	Unspecified	50–64 years (mean 58)	Blood, urine	Metabolomics, proteomics, genomics	EHRs; in person; remote assessment

a The cohorts presented in this table represent the 14 super cohorts with cardiovascular relevance identified and included in the review. Super cohorts are defined as cohorts with core features including systematic biosampling enabling biomarker and genetic analyses, linkage to national health records for complete outcome ascertainment and the availability of cardiovascular imaging, either across the full cohort or within large, well-characterized subsets. KOGES includes community-based studies (Ansan and Ansung), the Health Examinees (HEXA) study, and the Cardiovascular Disease Association Study (CAVAS). Age range, ethnicity, and sample size were obtained from official cohort profiles or publicly available study documentation where available; otherwise, these characteristics were extracted from peer-reviewed publications reporting large cohort subsets. As such, reported descriptive statistics may not precisely reflect the characteristics of the full cohort populations. Ethnicity is reported according to the definitions used by each cohort. Where a single ethnic group comprised more than two-thirds of participants, that group is reported as the predominant ethnicity. Country refers to the country in which recruitment was conducted. Sample size refers to the total number of participants recruited into each cohort. In the Hamburg City Health Study (HCHS), ‘other’ biosamples refer to tooth fluid and tonsil swabs. EHRs, electronic health records.

#### Imaging-focused cohorts

Fifty-seven cohorts (39%) incorporated cardiovascular imaging, enabling quantitative characterization of cardiac structure, function, and subclinical vascular disease (Table 2). The most frequent modalities were carotid ultrasound (54%) and echocardiography (47%), cardiac computed tomography angiography or coronary calcium scoring (28%), and cardiac magnetic resonance imaging (16%), with a small number of cohorts incorporating multiple imaging modalities.

**Table 2:** Longitudinal cohorts with cardiovascular and multiorgan imaging.^a^^,^^b^

Cohort name	Country	Sample size	ECG	Imaging coverage	CV imaging modality	Interval imaging	Multiorgan imaging
UK Biobank	UK	503 317	Y	Subset	Carotid ultrasound; CMR	Y	Brain MRI; abdominal MRI; DXA
German National Cohort (NAKO)	Germany	205 415	Y	Subset	Echocardiography; CMR	Y	Whole-body MRI; DXA
Hamburg City Health Study (HCHS)	Germany	45 000	Y	Subset	Echocardiography; carotid ultrasound; CMR; ultrasound aorta; Doppler ultrasound	Y	Brain MRI; optical CT
Gutenberg Health Study (GHS)	Germany	15 010	Y	Whole	Echocardiography; carotid ultrasound	N	OCT; retinal photography
Leipzig Research Centre for Civilization Diseases (LIFE)	Germany	16 994	Y	Whole	Echocardiography; carotid ultrasound; invasive coronary angiography	Y	Brain MRI
Paris Prospective Study III	France	10 157	N	Whole	Carotid ultrasound	N	N
Maastricht Study	Netherlands	9187	Y	Whole	Echocardiography; carotid ultrasound	N	Brain MRI; retinal photography; optical CT
Healthy Life in an Urban Setting Study (HELIUS)	Netherlands	24 789	Y	Subset	Carotid ultrasound; CMR; carotid MRI	N	Brain MRI
Nijmegen Exercise Study (NES)	Netherlands	25 000	N	Subset	Carotid ultrasound	N	N
Copenhagen General Population Study (CGPS)	Denmark	170 000	N	Subset	Cardiac CT	N	N
IBERICAN Study	Spain	8066	Y	Subset	Carotid ultrasound	N	N
Aragon Workers Health Study (AWHS)	Spain	5400	N	Subset	Carotid ultrasound; CT coronary calcium	Y	Abdominal ultrasound for vasculature
Corogene Study	Finland	5295	Y	Subset	Echocardiography; invasive coronary angiography	N	Chest radiography
Health 2000 Survey	Finland	9922	Y	Subset	Carotid ultrasound	N	N
Swedish Cardiopulmonary BioImage Study (SCAPIS)	Sweden	30 154	Y	Whole	Carotid ultrasound; CMR; cardiac CT	Y	Chest CT; liver ultrasound
Multi-Ethnic Study of Atherosclerosis (MESA)	USA	6814	Y	Subset	Echocardiography; carotid ultrasound; CMR; CT coronary calcium; carotid MRI	Y	Brain MRI
BioImage Study	USA	5808	N	Subset	Cardiac CT	N	N
St. Jude Lifetime Cohort (SJLIFE)	USA	8192	Y	Subset	Echocardiography	N	DXA; lumbar QCT; mammogram; breast MRI; colonoscopy
Emory Cardiovascular Biobank (EmCAB)	USA	6857	Y	Whole	Invasive coronary angiography	N	Chest radiography
Genetic Epidemiology of COPD (COPDGene)	USA	10 718	N	Whole	CT coronary calcium	N	Non-contrast chest CT
Cleveland Clinic GeneBank Study	USA	9880	Y	Whole	Coronary angiography; Coronary CT angiography	N	N
Dallas Heart Study (DHS)	USA	6101	Y	Subset	CMR; CT coronary calcium; carotid MRI; Aorta MRI	Y	Full-body MRI; MEG; DXA; liver proton MR spectroscopy
Jackson Heart Study	USA	5306	Y	Subset	Echocardiography; carotid ultrasound; CMR; cardiac CT; CT aorta	Y	CT abdomen
TRANSLATE-ACS Study	USA	12 243	Y	Whole	Coronary angiography	N	N
Health eHeart (HeH) Study	USA	214 097	Y	Subset	Echocardiography	Y	Multiorgan MRI
Brazilian Longitudinal Study of Adult Health (ELSA-Brasil)	Brazil	15 105	Y	Subset	Echocardiography; carotid ultrasound; CT coronary calcium	Y	Liver ultrasound; abdominal fat ultrasound; retinal photography
Canadian Longitudinal Study on Aging (CLSA)	Canada	51 338	Y	Subset	Carotid ultrasound	Y	DXA; retinal photography
CARTaGENE Study	Canada	43 000	Y	Subset	CMR	N	Brain MRI
Ontario Health Study (OHS)	Canada	225 620	N	Subset	CMR; carotid MRI	N	Brain MRI; abdominal MRI
Kailuan Study (KLS)	China	101 510	Y	Subset	Carotid ultrasound	N	Brain MRI
Guangzhou Biobank Cohort Study (GBCS)	China	30 430	Y	Subset	Echocardiography; carotid ultrasound	N	N
Heart Failure Registry of Patient Outcomes (HERO)	China	5620	Y	Subset	Echocardiography	N	N
Heart and Brain Investigation in Taicang Study (HABIT)	China	10 357	N	Y	Carotid ultrasound	Y	Transcranial sonography
China Pulmonary Thromboembolism Registry Study (CURES)	China	14 937	Y	Subset	Echocardiography; CT PA	N	N
Northeast China Rural Cardiovascular Health Study (NCRCHS)	China	11 956	Y	Subset	Echocardiography	N	N
China National Stroke Registry (CNSR)	China	62 066	Y	Subset	Echocardiography	N	Brain MRI; brain CT
China Elderly Degenerative Valve Disease Study (China-DVD)	China	8929	Y	Whole	Echocardiography	Y	N
No name—Community-based cohort from Jiading District	China	10 375	Y	Whole	Carotid ultrasound	N	Liver ultrasound
Namwon Study	South Korea	10 667	Y	Subset	Carotid ultrasound	Y	DXA; QUS; Chest radiography
Dong-gu Study (DGS)	South Korea	9260	Y	Subset	Echocardiography; carotid ultrasound	N	DXA; Retinogram; knee x-ray
Cardiovascular and Metabolic Diseases Etiology Research Center cohort (CMERC)	South Korea	8097	Y	Whole	Carotid ultrasound	N	N
Korean Acute Myocardial Infarction National Registry (KAMIR)	South Korea	86 118	Y	Whole	Echocardiography; invasive coronary angiography	N	N
Kangbuk Samsung Health Study (KSHS)	South Korea	290 000	N	Subset	Echocardiography; CT coronary calcium	N	Transcranial Doppler; abdominal ultrasound; retinal photography
Korean Genome and Epidemiology Study (KoGES)	South Korea	211 705	Y	Subset	Echocardiography; carotid ultrasound	N	N
Nagahama Prospective Genome Cohort for Comprehensive Human Bioscience Study	Japan	9769	Y	Subset	Echocardiography; carotid ultrasound	N	Brain MRI/MRA; DXA; optical CT; retinal photography; knee x-ray/MRI; chest radiography
Chronic Heart Failure Analysis and Registry in the Tohoku District-2 (CHART-2)	Japan	10 219	Y	Subset	Echocardiography; coronary CT angiography	Y	N
Tohoku Medical Megabank Community Cohort Study	Japan	87 865	Y	Subset	Carotid ultrasound	N	Retinal photography; OCT; calcaneal ultrasound
Health for Life in Singapore (HELIOS)	Singapore	10 004	Y	Whole	Carotid ultrasound	N	DXA; optical imaging (OCT/OCTA)
Qatar Biobank	Qatar	37 000	Y	Subset	Carotid ultrasound	N	DXA; retinal photography; whole-body MRI
LoCARPoN Study	India	7505	Y	Subset	Echocardiography; carotid ultrasound	N	Brain MRI
BioHEART-CT Study	Australia and New Zealand	5000	N	Whole	Coronary CT angiography	N	N
Health Effects of Arsenic Longitudinal Study (HEALS)	Bangladesh	24 000	Y	Subset	Carotid ultrasound	N	N
Center for cArdiometabolic Risk Reduction in South Asia (CARRS)	India, Pakistan	30 847	Y	Subset	Echocardiography; carotid ultrasound; CT coronary calcium	N	CT abdomen
EuroHeart Survey on Atrial Fibrillation	International	5333	Y	Subset	Echocardiography	N	N
EPICOR Study	International	23 573	Y	Whole	Invasive coronary angiography	N	N
RecordAF	International	5604	Y	Subset	Echocardiography	N	N
CONFIRM Registry	International	27 125	N	Whole	Coronary CT angiography; CT coronary calcium	N	N

a The table summarizes 57 longitudinal population-based cohorts included in this review that incorporate cardiovascular imaging. Sample size refers to the total number of participants recruited into each parent cohort. LIFE, Leipzig Research Centre for Civilization Diseases Cohort comprises the LIFE-Adult and LIFE-Heart cohorts only.

b Abbreviations: AF, atrial fibrillation; ASL, arterial spin labelling; CMR, cardiovascular magnetic resonance; CT PA, computed tomography pulmonary angiography; CT, computed tomography; CV, cardiovascular; DTI, diffusion tensor imaging; DXA, dual-energy X-ray absorptiometry; ECG, electrocardiography; ICA, invasive coronary angiography; IVUS, intravascular ultrasound; LoCARPON, Longitudinal Cognition and Aging Research on Population of the National capital region; MEG, magnetoencephalography; MRA, magnetic resonance angiography; MRI, magnetic resonance imaging; N, no; NA, North America; OCT, optical coherence tomography; OCTA, optical coherence tomography angiography; QCT, quantitative computed tomography; QUS, quantitative ultrasound; rs-fMRI, resting-state functional magnetic resonance imaging; SA, South America; SWI, susceptibility-weighted imaging; TCD, transcranial Doppler; TOF MRA, time-of-flight magnetic resonance angiography; US, ultrasound; Y, yes.

Phenotypic depth was further enhanced by interval imaging (28% of cohorts with cardiovascular imaging) or the addition of multi-organ evaluation (58%), offering unique insights into trajectories of cardiovascular remodelling and links between exposures, subclinical changes, and subsequent clinical outcomes.

#### Biosampling and omics-based phenotyping

Biosampling was performed in 133 cohorts (90%), universally including blood collection, and a smaller number collecting urine (48%), saliva (11%), stool (10%), hair (4%), and nail tissue (2%). Biomarker profiling was available in 80 cohorts (54%), ranging from conventional cardiometabolic risk markers to high-throughput platforms including proteomics and metabolomics.

Genetic data, obtained through genotyping or sequencing, were available in 74 cohorts (50%). The depth of genetic profiling varied considerably. Many cohorts employed genome-wide genotyping arrays with imputation to dense reference panels, whereas a smaller number incorporated more extensive whole-exome or whole-genome sequencing. Several resources combined genetic data with extensive phenotyping and longitudinal follow-up, enabling genome-wide association analyses, polygenic risk scoring, and causal inference approaches such as Mendelian randomization. In some cohorts, genetics were complemented by biomarker profiling, creating integrated genomics–phenomics platforms allowing analyses linking genetic variation to intermediate biological traits and clinical outcomes.

#### Wearable digital health technologies

Twenty-four cohorts (16%) incorporated wearable digital health technologies, most commonly accelerometery/actigraphy and heart rate or ECG rhythm monitoring to capture physical activity, cardiac rhythm, and sleep parameters (Supplementary Table S4). Technologies included wrist/hip/waist/thigh-worn accelerometers and Holter/ECG patch monitors, although some cohorts captured data via mobile applications (e.g. *Cardiogram* in the Health eHeart study [26]) or a home sleep screening device (ApneaLink in the Busselton Healthy Ageing Study [27]).

The All of Us Research Program uses a hybrid model combining investigator-provided devices with a ‘Bring Your Own Device’ pathway, enabling participants to share data from their personal devices [28]. Similarly, the Health eHeart Study, described as an ‘e-Cohort,’ uses a digital health framework to recruit and follow participants remotely, documenting self-reported exposures and outcomes, as well as integrating wearable-derived data to enable large-scale, real-world phenotyping beyond conventional clinic-based assessment [6, 29].

### Data harmonization

Substantial heterogeneity was observed across cohorts in recruitment strategies, phenotyping approaches, and follow-up and outcome ascertainment, which can limit comparability.

The most prominent cross-cohort harmonization initiative identified was the international ‘Health and Retirement Study’ umbrella, which supports consistent variable definitions and comparable analyses across numerous cohorts and study waves [30–36]. Other similar initiatives include the Cohorts for Heart and Aging Research in Genomic Epidemiology (CHARGE) consortium [37], which coordinates phenotype definitions to support genetics-based analyses across multiple population cohorts, including Age, Gene/Environment Susceptibility-Reykjavik (AGES-Reykjavik) [38], as well as the Biobank Standardisation and Harmonisation for Research Excellence in the European Union (BioSHaRE) project [39], which has performed harmonization of selected variables across eight European population-based studies.

## Discussion

This review systematically and comprehensively maps the global landscape of contemporary cohorts with cardiovascular relevance, highlighting key advances and gaps in representation and data modalities. This structured overview of cohort characteristics, design, and phenotyping strategies offers a practical resource to support cohort discovery and informed selection for cardiovascular research. This is particularly relevant given that cohort identification remains fragmented with inconsistent methodological reporting. We identified 147 large cohorts established since the year 2000, reflecting a rapid expansion of infrastructure that is moving cardiovascular epidemiology into an era where widespread replication, external validation, and cross-population comparisons are increasingly feasible.

A central finding is the persistent imbalance in global representation. Most cohorts were based in East Asia, Europe, and North America, whereas Africa, South America, the Middle East, and parts of Southeast Asia remain under-represented relative to population size. This may limit generalizability as many widely used risk models, imaging biomarkers, and omics-based discoveries are derived from high-income populations with distinct environmental exposures, healthcare systems, and background risk profiles. Notably, the most deeply phenotyped resources were also concentrated in these settings, potentially widening disparities in the global evidence base.

We also observed significant heterogeneity in cohort design. Population-based and clinical cohorts each offer distinct strengths, with the former facilitating discovery, risk prediction, and prevention research, whereas the latter often support more granular mechanistic investigation. However, this diversity complicates direct cross-cohort comparison and limits extrapolation across settings. Similarly, whilst multi-wave recruitment can enhance sample size and representation, differences between waves in participant characteristics, recruitment, phenotyping, and outcome ascertainment require detailed documentation and consideration of wave-adjusted analyses.

The apparent decline in cohorts in the 2020s should be interpreted cautiously. More recently established cohorts had less time to meet our required recruitment criteria, publish methodological descriptions, and become indexed in bibliographic databases. The present review therefore cannot determine whether this pattern reflects a genuine decline in new cohort establishment or, at least in part, publication and indexing lag.

Representativeness remains an important challenge. Many contemporary ‘population-based’ cohorts are volunteer cohorts with low response rates and systematic differences between participants and non-participants, creating potential for healthy volunteer and collider bias [40–43]. Such cohorts are not suitable for establishing population prevalence or incidence, although exposure–outcome associations are mostly reliable [40]. Some cohorts addressed this by using stratified or probability-based sampling or by oversampling historically under-represented groups [24, 44–46]. Transparent reporting of recruitment, response, and retention, as well as analytical strategies to reduce such biases, are key [47, 48].

Technological change has shifted population-based studies from traditional epidemiological evaluation towards integrated, multi-domain phenotyping, and scalable follow-up. Remote consent, app-based surveys, wearables, and electronic healthcare record linkage allow more scalable and granular data capture than traditional models. Biomarker and genetic data substantially enhance analytical potential, linking molecular pathways to clinical phenotypes, while wearables provide real-world data beyond episodic clinic-based assessments. In parallel, health record and registry linkage enables near-complete outcome capture and more standardized disease definitions (e.g. through International Classification of Disease or Observational Medical Outcomes Partnership Common Data Model [49] frameworks). Whilst these approaches can reduce burden and cost, they risk excluding individuals with limited digital access or literacy, introducing selection bias. Their adoption should therefore be accompanied by explicit attention to fairness and transparent governance.

Imaging-focused cohorts represent a cornerstone of contemporary cardiovascular research, with approximately one-third of cohorts incorporating cardiovascular imaging. By providing quantitative, continuous measures of structure and function, imaging enables assessment across the spectrum from early subclinical phenotypes to overt, clinically evident disease, supporting mechanistic insights and risk stratification. The concurrent availability of multi-modal, interval, and multi-organ imaging supports cross-modality validation, comprehensive cardiovascular phenotyping, longitudinal modelling, and integrated assessment of interactions between the cardiovascular system and other organ systems. Automated and artificial intelligence-based image analysis pipelines improve scalability and reproducibility, although harmonization of imaging and non-imaging data remains a key challenge.

Harmonization is essential to enable both comparative and pooled analyses. In practice, two complementary but distinct objectives can be considered: first, harmonization may aim to enable direct pooling of individual-level data across cohorts, requiring aligned definitions of exposures, covariates, and outcomes for combined analyses. Collaborations such as the Prospective Studies Collaboration and Emerging Risk Factors Collaboration demonstrate the scientific value of this approach, combining individual-level participant data across numerous studies to enable consistent analyses at a substantially greater scale. Second, harmonization may aim to define variables in a sufficiently comparable way to enable reliable comparison across independent cohorts, even without direct data pooling.

In this context, a distinction should be made between retrospective alignment and prospective harmonization. Retrospective alignment involves *post hoc* mapping of non-identical variables, often requiring assumptions and introducing heterogeneity. In contrast, prospective harmonization, through coordinated protocols, shared variables, and common data models, enables more robust pooled analyses and cross-cohort comparability. Where individual-level data cannot be pooled, harmonized analyses performed separately within cohorts and subsequently meta-analysed provide an alternative, exemplified by collaborations such as CHARGE [37]. Federated workflows within Trusted Research Environments may increasingly facilitate this approach, although with added complexity and potential differences in results [50]. The systematic characterization provided by this review can support these collaborative efforts by identifying cohorts with compatible populations and data domains for pooling, replication, or federated analyses.

The largest and most deeply phenotyped cohorts provide a strong foundation for integrative cardiovascular research, supporting development and validation of imaging- and omics-based biomarkers in diverse healthcare systems. They also offer optimal settings for the development of cross-cohort harmonization frameworks, including common exposure and endpoint definitions, and reproducible analysis pipelines.

Existing online discovery platforms provide complementary resources, with broad catalogues of studies and information on access, governance, and data availability. In contrast, this review systematically identifies a specifically defined subgroup of contemporary longitudinal cohorts relevant to cardiovascular research, applying a standardized framework to compare their design and phenotypic content. In particular, we systematically characterize cardiovascular imaging availability, modality, coverage, interval imaging, and accompanying multi-organ imaging, which is not captured by existing discovery platforms. Differences in cohort coverage between these resources may therefore reflect differences in scope, eligibility criteria, and purpose. Future complementary work may examine scientific output, impact, and policy influence of the identified cohorts to highlight characteristics associated with particularly productive or influential resources.

### Strengths and limitations

This review has several strengths. We used systematic searches across multiple databases, including GIM to improve ascertainment from under-represented regions and applied consistent eligibility criteria. Standardized extraction enabled direct comparison across multiple phenotypic domains. The resulting searchable resource may facilitate cohort discovery, cross-cohort replication, and large-scale collaborative research.

Several limitations of this review should be acknowledged. We relied on published reports and publicly available documentation. Incomplete reporting may have led to misclassification or underestimation of available data modalities. We did not perform a formal risk-of-bias or methodological quality assessment of individual cohorts, as the objective was descriptive mapping rather than evaluation of internal validity.

## Conclusion

Contemporary longitudinal cohorts with cardiovascular relevance provide multi-modal, integrative epidemiology at scale. Persistent geographic under-representation and heterogeneity in recruitment, phenotyping, and follow-up remain key barriers to generalizability. Future progress will depend on broader representation, improved reporting transparency, and harmonization to maximize the scientific value of available resources.

## Ethics approval

No new individual-level data were generated or analysed in support of this research, and specific ethical approval was therefore not sought.

## Supplementary Material

dyag241_Supplementary_Data

## Data Availability

No new individual-level data were generated or analysed in support of this research. The study is based exclusively on information extracted from publicly available sources and published reports, which are cited within the article.
