# Supplementary material for: The global landscape of contemporary longitudinal cohorts for cardiovascular research: a systematic review
Source: Int J Epidemiol. 2026 Nov 16;55(6):dyag241. doi: 10.1093/ije/dyag241 (PMC13770544; doi:10.1093/ije/dyag241)
Supplement: Supplementary file 1 [file dyag241_supplementary_data.zip › ije-2026-05-0920-File009.docx]

**The Global Landscape of Contemporary Longitudinal Cohorts for Cardiovascular Research**

**Supplementary Material**

Table of Contents

[Supplementary Methods	2](#_Toc227254734)

[Supplementary Table 1. PRISMA-S checklist	4](#_Toc227254735)

[Supplementary Table 2. Data extraction spreadsheet	7](#_Toc227254736)

[Supplementary Table 3. Overview of all eligible longitudinal cardiovascular cohorts	9](#_Toc227254737)

[Supplementary Table 4. Longitudinal cardiovascular cohorts incorporating wearable digital health data	10](#_Toc227254738)

[Supplementary References	12](#_Toc227254739)

# **Supplementary Methods**

**Search strategy**

We performed searches of MEDLINE (via PubMed) and Embase to identify longitudinal cohort studies with cardiovascular relevance published after 1^st^ January 2000. Searches used controlled vocabulary terms (Medical Subject Headings [MeSH] in PubMed and equivalent Emtree terms in Embase) relating to cardiovascular disease, cohort or longitudinal study design, and adult populations.

To enhance visibility of under-represented regions, we also systematically searched the Global Index Medicus database (GIM). Searches combined cardiovascular subject headings and keywords with terms for longitudinal or cohort study design, using DeCS (Descriptors in Health Sciences)/MeSH descriptors adapted for GIM. Searches were restricted to English-language publications from 2000 onwards using a database-specific observational study filter. Additional eligible cohorts were identified through reference list screening of included articles and relevant reviews. Complete, line-by-line search strategies for all databases are provided below (including all limits and filters applied).

**Database search strings**

PubMed/MEDLINE search

*Search date: 4^th^ March 2025*

"Cardiovascular Diseases"[Majr] AND "Adult"[Mesh] AND "Longitudinal Studies"[MeSH] AND “Cohort Studies”[MeSH]. Search results restricted to 2000 onwards.

Embase search

*Search date: 4^th^ March 2025*

'cardiovascular disease'/exp/mj AND ‘adult'/exp AND 'longitudinal study'/exp AND 'cohort analysis'/exp AND (2000:py OR 2001:py OR 2002:py OR 2003:py OR 2004:py OR 2005:py OR 2006:py OR 2007:py OR 2008:py OR 2009:py OR 2010:py OR 2011:py OR 2012:py OR 2013:py OR 2014:py OR 2015:py OR 2016:py OR 2017:py OR 2018:py OR 2019:py OR 2020:py OR 2021:py OR 2022:py OR 2023:py OR 2024:py OR 2025:py).

Global Index Medicus (GIM) search

*Search date: 20^th^ August 2025*

mh:("Cardiovascular Diseases") AND (type_of_study:("observational_studies")) AND (year_cluster: [2000 TO 2025]) AND (la:("en")).

Cross-referencing

In addition to the database searches, reference lists of included cohort profile publications and relevant review articles were manually screened to identify additional eligible cohorts not captured through the electronic search strategy

**Detailed eligibility criteria**

Publications were screened to identify underlying cohorts used for analysis, and overall cohort characteristics were assessed for eligibility. Cohorts were eligible if they met all of the following criteria:

(1) exclusive recruitment of adults aged ≥18 years;

(2) start of recruitment from the year 2000 onwards;

(3) minimum sample size of 5,000 participants recruited in the first recruitment wave;

(4) prospective longitudinal cohort design, defined as one or more planned or completed follow-up assessments ≥12 months after recruitment, based on active, passive follow-up, or both;

(5) cardiovascular focus or relevance, defined as collection of cardiovascular exposures and/or outcomes, without requiring the cohort to be exclusively cardiovascular in scope.

Excluded were studies that were purely retrospective or cross-sectional, interventional studies, and post-hoc analyses of clinical trial populations that were not designed prospectively as longitudinal cohorts. We also excluded registry-only or insurance-claims-based sources without study-specific consent, assessments, or follow-up. Studies with unclear methodology or insufficient publicly available information to assess eligibility were excluded.

**Cohort selection**

All records identified through database searches were downloaded from each search platform in tabular format and imported into Microsoft Excel, where results were collated and duplicates were removed. Abstracts were screened by four reviewers (D.G.C., M.R., R.R. and J.S.) to identify potentially eligible cohorts according to the above criteria. Each cohort was subsequently assessed in detail by a second reviewer, who cross-checked the eligibility assessment against the available source material. The second reviewer had access to the first reviewer’s assessment, and this was therefore a verification step rather than an independent second eligibility assessment. Any discrepancies were resolved through discussion and consensus, with arbitration by senior investigators (Z.R-E and A.J.L.).

**Data extraction**

Data were extracted using a predesigned, standardised data extraction template developed by the study team (**Supplementary Table 2**). The template captured: (1) general cohort characteristics (recruitment countries, sample size, details regarding recruitment); (2) details of the participant sample (age, sex, ethnicity); and (3) available data domains, including physical measures, lifestyle/environmental factors, medical history/medication use, biosampling, cognitive assessments, cardiovascular/multiorgan imaging, electrocardiogram (ECG), wearables, omics and genetic data.

To distinguish study ‘waves’ from serial reassessment of the same participants, recruitment waves were explicitly characterised as the recruitment of new, study-naive participants into the same cohort. Where rolling recruitment occurred with no interruption, this was considered as one single recruitment wave. Smaller pilot phases preceding the main rollout of the cohort were not classified as separate waves.

For cardiovascular imaging, we recorded whether availability was in the full cohort or subsets of participants, the imaging modality, and the presence of interval imaging, where applicable. Follow-up characteristics, including follow-up type (active, passive or mixed) and mechanisms (face-to-face assessment, remote assessment, health record linkage or other), were documented.

## **Supplementary Table 1. PRISMA-S checklist**

| **Section and Topic** | **Item #** | **Checklist item** | **Location where item is reported** |
| --- | --- | --- | --- |
| **TITLE** | | |  |
| Title | 1 | Identify the report as a systematic review. | Page 1 |
| **ABSTRACT** | | |  |
| Abstract | 2 | See the PRISMA 2020 for Abstracts checklist. | Page 3 |
| **INTRODUCTION** | | |  |
| Rationale | 3 | Describe the rationale for the review in the context of existing knowledge. | Pages 5-6 |
| Objectives | 4 | Provide an explicit statement of the objective(s) or question(s) the review addresses. | Page 6 |
| **METHODS** | | |  |
| Eligibility criteria | 5 | Specify the inclusion and exclusion criteria for the review and how studies were grouped for the syntheses. | Page 7 |
| Information sources | 6 | Specify all databases, registers, websites, organisations, reference lists and other sources searched or consulted to identify studies. Specify the date when each source was last searched or consulted. | Page 7  Supplementary Page 2 |
| Search strategy | 7 | Present the full search strategies for all databases, registers and websites, including any filters and limits used. | Page 7  Supplementary Page 2 |
| Selection process | 8 | Specify the methods used to decide whether a study met the inclusion criteria of the review, including how many reviewers screened each record and each report retrieved, whether they worked independently, and if applicable, details of automation tools used in the process. | Page 8 |
| Data collection process | 9 | Specify the methods used to collect data from reports, including how many reviewers collected data from each report, whether they worked independently, any processes for obtaining or confirming data from study investigators, and if applicable, details of automation tools used in the process. | Page 8  Supplementary Table 2 |
| Data items | 10a | List and define all outcomes for which data were sought. Specify whether all results that were compatible with each outcome domain in each study were sought (e.g. for all measures, time points, analyses), and if not, the methods used to decide which results to collect. | Not applicable |
|  | 10b | List and define all other variables for which data were sought (e.g. participant and intervention characteristics, funding sources). Describe any assumptions made about any missing or unclear information. | Supplementary Table 2 |
| Study risk of bias assessment | 11 | Specify the methods used to assess risk of bias in the included studies, including details of the tool(s) used, how many reviewers assessed each study and whether they worked independently, and if applicable, details of automation tools used in the process. | Not applicable |
| Effect measures | 12 | Specify for each outcome the effect measure(s) (e.g. risk ratio, mean difference) used in the synthesis or presentation of results. | Not applicable |
| Synthesis methods | 13a | Describe the processes used to decide which studies were eligible for each synthesis (e.g. tabulating the study intervention characteristics and comparing against the planned groups for each synthesis (item #5)). | Pages 8-9 |
|  | 13b | Describe any methods required to prepare the data for presentation or synthesis, such as handling of missing summary statistics, or data conversions. | Page 8 |
|  | 13c | Describe any methods used to tabulate or visually display results of individual studies and syntheses. | Page 9 |
|  | 13d | Describe any methods used to synthesize results and provide a rationale for the choice(s). If meta-analysis was performed, describe the model(s), method(s) to identify the presence and extent of statistical heterogeneity, and software package(s) used. | Page 9 |
|  | 13e | Describe any methods used to explore possible causes of heterogeneity among study results (e.g. subgroup analysis, meta-regression). | Not applicable |
|  | 13f | Describe any sensitivity analyses conducted to assess robustness of the synthesized results. | Not applicable |
| Reporting bias assessment | 14 | Describe any methods used to assess risk of bias due to missing results in a synthesis (arising from reporting biases). | Not applicable |
| Certainty assessment | 15 | Describe any methods used to assess certainty (or confidence) in the body of evidence for an outcome. | Not applicable |
| **RESULTS** | | |  |
| Study selection | 16a | Describe the results of the search and selection process, from the number of records identified in the search to the number of studies included in the review, ideally using a flow diagram. | Page 10  Figure 1 PRISMA flow diagram  Graphical abstract |
|  | 16b | Cite studies that might appear to meet the inclusion criteria, but which were excluded, and explain why they were excluded. | Page 10  Figure 1 PRISMA flow diagram |
| Study characteristics | 17 | Cite each included study and present its characteristics. | Pages 10-19  Table 1-2, Supplementary Table 3-4 |
| Risk of bias in studies | 18 | Present assessments of risk of bias for each included study. | Not applicable |
| Results of individual studies | 19 | For all outcomes, present, for each study: (a) summary statistics for each group (where appropriate) and (b) an effect estimate and its precision (e.g. confidence/credible interval), ideally using structured tables or plots. | Not applicable |
| Results of syntheses | 20a | For each synthesis, briefly summarise the characteristics and risk of bias among contributing studies. | Pages 10-21; Table 1–2; Figures 2–3; Supplementary Tables 3–4.  No formal risk-of-bias assessment was performed. |
|  | 20b | Present results of all statistical syntheses conducted. If meta-analysis was done, present for each the summary estimate and its precision (e.g. confidence/credible interval) and measures of statistical heterogeneity. If comparing groups, describe the direction of the effect. | Not applicable |
|  | 20c | Present results of all investigations of possible causes of heterogeneity among study results. | Not applicable |
|  | 20d | Present results of all sensitivity analyses conducted to assess the robustness of the synthesized results. | Not applicable |
| Reporting biases | 21 | Present assessments of risk of bias due to missing results (arising from reporting biases) for each synthesis assessed. | Not applicable |
| Certainty of evidence | 22 | Present assessments of certainty (or confidence) in the body of evidence for each outcome assessed. | Not applicable |
| **DISCUSSION** | | |  |
| Discussion | 23a | Provide a general interpretation of the results in the context of other evidence. | Pages 22-27 |
|  | 23b | Discuss any limitations of the evidence included in the review. | Page 22-27 |
|  | 23c | Discuss any limitations of the review processes used. | Page 26-27 |
|  | 23d | Discuss implications of the results for practice, policy, and future research. | Pages 22-27 |
| **OTHER INFORMATION** | | |  |
| Registration and protocol | 24a | Provide registration information for the review, including register name and registration number, or state that the review was not registered. | Page 7 |
|  | 24b | Indicate where the review protocol can be accessed, or state that a protocol was not prepared. | Protocol available via PROSPERO  (1290508) |
|  | 24c | Describe and explain any amendments to information provided at registration or in the protocol. | Not applicable |
| Support | 25 | Describe sources of financial or non-financial support for the review, and the role of the funders or sponsors in the review. | Page 28 |
| Competing interests | 26 | Declare any competing interests of review authors. | Page 28 |
| Availability of data, code and other materials | 27 | Report which of the following are publicly available and where they can be found: template data collection forms; data extracted from included studies; data used for all analyses; analytic code; any other materials used in the review. | Page 28 |

**Footnote.** From: Page MJ, McKenzie JE, Bossuyt PM, Boutron I, Hoffmann TC, Mulrow CD, et al. The PRISMA 2020 statement: an updated guideline for reporting systematic reviews. BMJ 2021;372:n71. doi: 10.1136/bmj.n71. This work is licensed under CC BY 4.0. To view a copy of this license, visit https://creativecommons.org/licenses/by/4.0/

### **Supplementary Table 2. Data extraction spreadsheet**

| **Items** | **Notes** | **Coding (if applicable)** |
| --- | --- | --- |
| Cohort name | Name of the cohort study | Free text |
| Countries | Countries where participants were recruited | Free text |
| Sex distribution | Sex of recruited participants and, where available, proportion of males and females | Female / Male |
| Ethnicity | Cohorts were classified as multi-ethnic only if no single ethnic group accounted for more than two-thirds of participants; otherwise, the predominant ethnic group was recorded | Free text |
| Age range | Eligibility age range at recruitment | Free text |
| Sample size | Total number of participants recruited | Free text |
| Cohort type | Overall cohort design | Unselected population-based (regional / national / international); Clinical; Occupational; Other |
| Number recruited | Number of participants recruited in the first recruitment wave | Free text |
| Years recruited | Calendar years during which the first recruitment wave took place | Free text |
| > 1 recruitment wave? | Whether the cohort included more than one recruitment wave | Yes /No |
| Physical measures | Availability of physical measurements | Anthropometrics / Vital signs / Body composition / Lung function/Spirometry / Physical performance / Vision/Hearing / Arterial stiffness / Other |
| Environmental | Availability of environmental data | Geographic location / Air pollution / Other |
| Lifestyle | Availability of lifestyle data | Smoking / Alcohol / Diet/Nutrition / Physical activity / Sleep / Sexual/Reproductive factors / Electronic device use / Socioeconomic status / Other |
| Medical history | Whether medical history data were collected | Yes / No |
| Medication use | Whether medication data were collected | Yes / No |
| Biological samples | Availability of biological samples | Blood / Urine / Saliva / Sputum / Stool / Tissue / Other |
| Cognitive assessments | Availability of cognitive testing | Yes / No |
| ECG | Whether ECG data were collected | Free text |
| Wearables/ Holter's | Use of wearable devices or Holter monitoring | Free text |
| CV imaging | Whether CV-specific imaging was performed | Yes / No |
| Imaging - whole vs subset | Whether imaging was performed in the full cohort or a subset | Whole / Subset |
| CV imaging modality | Cardiovascular imaging modalities used | CT coronary calcium / CT coronary angiogram / Cardiac MRI / Echocardiography / Carotid ultrasound / Carotid MRI / Invasive coronary angiography / Other |
| Interval imaging | Availability of repeat (interval) imaging | Yes / No |
| Multiorgan imaging | Availability of imaging beyond the cardiovascular system | Free text |
| Biomarker profiling | Availability of biomarker analyses using omics-based techniques | Yes / No |
| Genetic data | Availability of genetic data | Yes / No |
| Follow-up type | Overall follow-up approach | Active / Passive / Mixed |
| Follow-up mechanism/method | Method of follow-up | Face-to-face; Remote assessment; Health record linkage; Other |
| Website | Cohort website | Link |

**Footnote.** This table represents the structured data extraction proforma used in an Excel-based data collection tool for eligible cohorts. Pre-specified coding categories (implemented via dropdown menus) were defined a priori by the study team. Where free-text fields were required, entries were reviewed and harmonised using consistent terminology across the four independent reviewers to ensure standardisation.

#### **Supplementary Table 3. Overview of all eligible longitudinal cardiovascular cohorts**

Online only, searchable database

##### **Supplementary Table 4. Longitudinal cardiovascular cohorts incorporating wearable digital health data**

| *Cohort name* | *Sample*  *size* | *Wearable device*  *type* | *Indication* | *Monitoring*  *duration* | *Planned/ published* |
| --- | --- | --- | --- | --- | --- |
| UK Biobank | 503,317 | Wrist-worn accelerometer (Axivity AX3) | Physical activity; Sleep | 7 days | Published |
| German National Cohort (NAKO) | 205,415 | Hip-worn accelerometer (ActiGraph);  SomnoWatch actigraphy | Physical activity; Sleep; Cardiac rhythm; Respiratory assessment | 7 days (ActiGraph); 24 h (SomnoWatch) | Published |
| Multi-Ethnic Study of Atherosclerosis (MESA) | 6,814 | Ambulatory ECG patch monitoring | Subclinical AF detection | Up to 14 days | Published |
| Brazilian Longitudinal Study of Adult Health (ELSA-Brasil) | 15,105 | Hip/waist-worn accelerometer (ActiGraph); Wrist actigraphy (Actiwatch) | Physical activity; Sleep | 7 days | Published |
| Busselton Healthy Ageing Study (BHAS) | 5,107 | Home sleep apnoea monitoring (ApneaLink) | Sleep-disordered breathing | 1 night | Published |
| Dallas Heart Study (DHS) | 6,101 | Wrist-worn Actical accelerometer | Physical activity | 7 days | Published |
| WHO Study on Global Ageing and Adult Health (SAGE) | 63,445 | Hip-worn accelerometer (ActiGraph) | Physical activity | 7 days | Published |
| Osteoporotic Fractures in Men Study (MrOS) | 5,994 | Wrist actigraphy (Sleep-Watch-O) | Sleep; Rest–activity rhythms | 5 days | Published |
| Hispanic Community Health Study–Study of Latinos (HCHS–SOL) | 16,415 | Wrist actigraphy (Actiwatch) | Sleep; Rest–activity rhythms | 7 days | Published |
| Maastricht Study | 9,187 | Thigh-worn accelerometer (ActivPAL3) | Physical activity | 8 days | Published |
| Leipzig Research Centre for Civilization Diseases (LIFE) cohorts | 16,994 | 2D-axial armband accelerometer; Ambulatory ECG | Physical activity; Sleep; Cardiac rhythm | 7 days | Published |
| Health eHeart Study (HeH) | 214,097 | Smartphone-based PPG app | Heart rate monitoring | Episodic | Published |
| National Center for Geriatric and Gerontology-Study of Geriatric Syndromes Cohort | 11,000 | Waist-worn accelerometer (ActiGraph) | Physical activity | 7 days | Published |
| Health 2000 Survey | 9,922 | Hip-worn accelerometer (ActiGraph) | Physical activity | 7 days | Published |
| Tsuruoka Metabolomics Cohort Study (TMCS) | 11,002 | Waist/hip-worn accelerometer (ActiGraph) | Physical activity | 7 days | Published |
| Nagahama Prospective Genome Cohort for Comprehensive Human Bioscience Study | 9,769 | Wrist actigraphy (Actiwatch); Nocturnal pulse oximetry (PULSOX-Me300) | Sleep; Sleep-disordered breathing | ≥2 nights | Published |
| Healthy Finland Survey | 28,153 | Wrist-worn accelerometer | Physical activity; Sleep | 7 days | Published |
| Health for Life in Singapore (HELIOS) | 10,004 | Wrist-worn accelerometer | Physical activity | 7 days | Published |
| Nijmegen Exercise Study (NES) | 25,000 | Thigh-worn accelerometer (ActivPAL3) | Physical activity; Sedentary behaviour/posture; Sleep (derived) | 8 days | Published |
| Tohoku Medical Megabank Community Cohort | 87,865 | Waist-worn pedometer | Physical activity | 14 days | Published |
| All of Us | 849,000 | Wrist-worn wearable (Fitbit; accelerometer + PPG) | Physical activity; Heart rate monitoring; Sleep | Variable | Published |
| Hamburg City Health Study | 45,000 | Wrist-worn accelerometer (ActiGraph) | Physical activity | 7 days | Planned |
| Center for cArdiometabolic Risk Reduction in South Asia (CARRS) | 30,847 | Wrist actigraphy (Actiwatch) | Sleep; Total steps | 7 days | Planned |
| Our Future Health (OFH) | 2,461,601 | Fitness tracker; Smartwatch data | Physical activity | Variable | Planned |

**Footnote.** The table summarises wearable digital health data from the 24 longitudinal cohorts included in this systematic review. Sample size refers to the total number of participants recruited into each cohort. Wearable data were collected in a subset of participants in all listed cohorts. LIFE, Leipzig Research Centre for Civilization Diseases Cohort comprises the LIFE-Adult and LIFE-Heart cohorts only. **Abbreviations**: AF, atrial fibrillation; PPG, photoplethysmography; ECG, electrocardiography; RecordAF, Registry on Cardiac rhythm disorders assessing the control of Atrial Fibrillation.

###### **Supplementary References**

References correspond to the cohorts listed in **Supplementary Table 3** and are presented in the same order. Where available, we cite the primary cohort profile publication or peer-reviewed article describing the cohort design. If no peer-reviewed publication was available, the official cohort website was cited.

1. Sudlow C, Gallacher J, Allen N, Beral V, Burton P, Danesh J, et al. UK Biobank: An Open Access Resource for Identifying the Causes of a Wide Range of Complex Diseases of Middle and Old Age. PLoS Med. 2015 Mar 31;12(3):e1001779. doi:10.1371/journal.pmed.1001779
2. Perianayagam A, Bloom D, Lee J, Parasuraman S, Sekher T V, Mohanty SK, et al. Cohort Profile: The Longitudinal Ageing Study in India (LASI). Int J Epidemiol. 2022 Aug 10;51(4):e167–76. doi:10.1093/ije/dyab266
3. Kweon SS, Shin MH, Jeong SK, Nam HS, Lee YH, Park KS, et al. Cohort Profile: The Namwon Study and the Dong-gu Study. Int J Epidemiol. 2014 Apr 1;43(2):558–67. doi:10.1093/ije/dys244
4. Leitsalu L, Haller T, Esko T, Tammesoo ML, Alavere H, Snieder H, et al. Cohort Profile: Estonian Biobank of the Estonian Genome Center, University of Tartu. Int J Epidemiol. 2015 Aug;44(4):1137-47. doi: 10.1093/ije/dyt268
5. Korea Employment Information Service (KEIS). Korean longitudinal study of ageing (KLoSA) (2015). Available at: https://survey.keis.or.kr/eng/klosa/klosa01.jsp (Accessed 15 April 2026).
6. Blaha MJ, DeFilippis AP. Multi-Ethnic Study of Atherosclerosis (MESA). J Am Coll Cardiol. 2021 Jun;77(25):3195–216. doi:10.1016/j.jacc.2021.05.006
7. Sarrafzadegan N, Talaei M, Sadeghi M, Kelishadi R, Oveisgharan S, Mohammadifard N, et al. The Isfahan cohort study: Rationale, methods and main findings. J Hum Hypertens. 2011 Sep 25;25(9):545–53. doi:10.1038/jhh.2010.99
8. Schmidt MI, Duncan BB, Mill JG, Lotufo PA, Chor D, Barreto SM, et al. Cohort Profile: Longitudinal Study of Adult Health (ELSA-Brasil). Int J Epidemiol. 2015 Feb 1;44(1):68–75. doi:10.1093/ije/dyu027
9. Zhang Q, Zhou Y, Gao X, Wang C, Zhang S, Wang A, et al. Ideal Cardiovascular Health Metrics and the Risks of Ischemic and Intracerebral Hemorrhagic Stroke. Stroke. 2013 Sep;44(9):2451–6. doi:10.1161/STROKEAHA.113.678839
10. Ruggiero E, Di Castelnuovo A, Costanzo S, Esposito S, De Curtis A, Persichillo M, et al. Olive oil consumption is associated with lower cancer, cardiovascular and all-cause mortality among Italian adults: prospective results from the Moli-sani Study and analysis of potential biological mechanisms. Eur J Clin Nutr. 2024 Aug;78(8):684-693. doi: 10.1038/s41430-024-01442-8
11. Donoghue OA, McGarrigle CA, Foley M, Fagan A, Meaney J, Kenny RA. Cohort Profile Update: The Irish Longitudinal Study on Ageing (TILDA). Int J Epidemiol. 2018 Oct 1;47(5):1398–1398l. doi:10.1093/ije/dyy163
12. Raina P, Wolfson C, Kirkland S, Griffith LE, Balion C, Cossette B, et al. Cohort Profile: The Canadian Longitudinal Study on Aging (CLSA). Int J Epidemiol. 2019 Dec 1;48(6):1752–1753j. doi:10.1093/ije/dyz173
13. Zhao Y, Hu Y, Smith JP, Strauss J, Yang G. Cohort Profile: The China Health and Retirement Longitudinal Study (CHARLS). Int J Epidemiol. 2014 Feb 1;43(1):61–8. doi:10.1093/ije/dys203
14. Peters A, Peters A, Greiser KH, Göttlicher S, Ahrens W, Albrecht M, et al. Framework and baseline examination of the German National Cohort (NAKO). Eur J Epidemiol. 2022 Oct 19;37(10):1107–24. doi:10.1007/s10654-022-00890-5
15. Kweon SS, Shin MH, Jeong SK, Nam HS, Lee YH, Park KS, et al. Cohort Profile: The Namwon Study and the Dong-gu Study. Int J Epidemiol. 2014 Apr 1;43(2):558–67. doi:10.1093/ije/dys244
16. Mortensen MB, Afzal S, Nordestgaard BG, Falk E. The high-density lipoprotein-adjusted SCORE model worsens SCORE-based risk classification in a contemporary population of 30 824 Europeans: the Copenhagen General Population Study. Eur Heart J. 2015 Sep 21;36(36):2446–53. doi:10.1093/eurheartj/ehv251
17. Rannan-Eliya RP, Dissanayake VH, Perera P, Perera B, Herath HMM, Wijemunige N, et al. Cohort Profile: The Sri Lanka Health and Ageing Study (SLHAS). Int J Epidemiol. 2024 Feb 14;53(2). doi:10.1093/ije/dyae044
18. Shim JS, Song BM, Lee JH, Lee SW, Park JH, Choi DP, et al. Cardiovascular and Metabolic Diseases Etiology Research Center (CMERC) cohort: study protocol and results of the first 3 years of enrollment. Epidemiol Health. 2017 Apr 1;39:e2017016. doi:10.4178/epih.e2017016
19. Awadalla P, Boileau C, Payette Y, Idaghdour Y, Goulet JP, Knoppers B, et al. Cohort profile of the CARTaGENE study: Quebec’s population-based biobank for public health and personalized genomics. Int J Epidemiol. 2013 Oct;42(5):1285–99. doi:10.1093/ije/dys160
20. Kotov A, Peters F, Debus ES, Zeller T, Heider P, Stavroulakis K, et al. The prospective GermanVasc cohort study. Vasa. 2021 Nov;50(6):446-452.
21. Jiang CQ, Lam TH, Lin JM, Liu B, Yue XJ, Cheng KK, et al. An overview of the Guangzhou Biobank Cohort Study–Cardiovascular Disease Subcohort (GBCS-CVD): a platform for multidisciplinary collaboration. J Hum Hypertens. 2010 Feb 9;24(2):139–50. doi:10.1038/jhh.2009.52
22. Steptoe A, Breeze E, Banks J, Nazroo J. Cohort Profile: The English Longitudinal Study of Ageing. Int J Epidemiol. 2013 Dec 1;42(6):1640–8. doi:10.1093/ije/dys168
23. Howell CR, Bjornard KL, Ness KK, Alberts N, Armstrong GT, Bhakta N, et al. Cohort Profile: The St. Jude Lifetime Cohort Study (SJLIFE) for paediatric cancer survivors. Int J Epidemiol. 2021 Mar 3;50(1):39–49. doi:10.1093/ije/dyaa203
24. Howard VJ, Cushman M, Pulley L, Gomez CR, Go RC, Prineas RJ, et al. The Reasons for Geographic and Racial Differences in Stroke Study: Objectives and Design. Neuroepidemiology. 2005;25(3):135–43. doi:10.1159/000086678
25. Feng YCA, Chen CY, Chen TT, Kuo PH, Hsu YH, Yang HI, et al. Taiwan Biobank: A rich biomedical research database of the Taiwanese population. Cell Genomics. 2022 Nov;2(11):100197. doi:10.1016/j.xgen.2022.100197
26. Sakata Y, Nochioka K, Miura M, Tadaki S, Ushigome R, Yamauchi T, et al. The Chronic Heart Failure Analysis and Registry in the Tohoku District (CHART) Study. J Card Fail. 2014 Oct;20(10):S137. doi:10.1016/j.cardfail.2014.07.028
27. Anantanasuwong D, Theerawanviwat D, Siripanich P. Panel Survey and Study on Health, Aging, and Retirement in Thailand. In: Encyclopedia of Gerontology and Population Aging. Cham: Springer International Publishing; 2019. p. 1–6. doi:10.1007/978-3-319-69892-2_982-1
28. James A, Hunter M, Straker L, Beilby J, Bucks R, Davis T, et al. Rationale, design and methods for a community-based study of clustering and cumulative effects of chronic disease processes and their effects on ageing: the Busselton healthy ageing study. BMC Public Health. 2013 Dec 8;13(1):936. doi:10.1186/1471-2458-13-936
29. Victor RG, Haley RW, Willett DL, Peshock RM, Vaeth PC, Leonard D, et al. The Dallas Heart Study: a population-based probability sample for the multidisciplinary study of ethnic differences in cardiovascular health. Am J Cardiol. 2004 Jun;93(12):1473–80. doi:10.1016/j.amjcard.2004.02.058
30. German Cancer Research Center (DKFZ). ESTHER study: Epidemiological study on potentials for prevention, early detection and optimized treatment of chronic diseases in the elderly population. <https://www.dkfz.de/en/clinical-epidemiology-of-early-cancer-detection/esther> (accessed 16 April 2026).
31. Ohsawa M, Itai K, Tanno K, Onoda T, Ogawa A, Nakamura M, et al. Cardiovascular risk factors in the Japanese northeastern rural population. Int J Cardiol. 2009 Nov;137(3):226–35. doi:10.1016/j.ijcard.2008.06.052
32. Jagodzinski A, Johansen C, Koch-Gromus U, Aarabi G, Adam G, Anders S, et al. Rationale and Design of the Hamburg City Health Study. Eur J Epidemiol. 2020 Feb 8;35(2):169–81. doi:10.1007/s10654-019-00577-4
33. Zheng L, Zhang Z, Sun Z, Li J, Zhang X, Xu C, et al. The association between body mass index and incident hypertension in rural women in China. Eur J Clin Nutr. 2010 Aug 9;64(8):769–75. doi:10.1038/ejcn.2010.97
34. Cinza-Sanjurjo S, Prieto-Díaz MA, Pallares-Carratala V, Mico-Pérez RM, Barquilla-García A, Velilla-Zancada SM, et al. Prevalence of obesity in primary care patients: The IBERICAN study. Nutrition, Metabolism and Cardiovascular Diseases. 2025 May;35(5):103751. doi:10.1016/j.numecd.2024.09.021
35. Pourshams A, Khademi H, Malekshah AF, Islami F, Nouraei M, Sadjadi AR, et al. Cohort Profile: The Golestan Cohort Study--a prospective study of oesophageal cancer in northern Iran. Int J Epidemiol. 2010 Feb 1;39(1):52–9. doi:10.1093/ije/dyp161
36. Rossi MCE, Lucisano G, Comaschi M, Coscelli C, Cucinotta D, Di Blasi P, et al. Quality of Diabetes Care Predicts the Development of Cardiovascular Events: Results of the AMD-QUASAR Study. Diabetes Care. 2011 Feb 1;34(2):347–52. doi:10.2337/dc10-1709
37. Wong R, Michaels-Obregon A, Palloni A. Cohort Profile: The Mexican Health and Aging Study (MHAS). Int J Epidemiol. 2017 Apr 1;46(2):e2–e2. doi:10.1093/ije/dyu263
38. Teo K, Chow CK, Vaz M, Rangarajan S, Yusuf S. The Prospective Urban Rural Epidemiology (PURE) study: Examining the impact of societal influences on chronic noncommunicable diseases in low-, middle-, and high-income countries. Am Heart J. 2009 Jul;158(1):1-7.e1. doi:10.1016/j.ahj.2009.04.019
39. Kowal P, Chatterji S, Naidoo N, Biritwum R, Fan W, Lopez Ridaura R, et al. Data Resource Profile: The World Health Organization Study on global AGEing and adult health (SAGE). Int J Epidemiol. 2012 Dec 1;41(6):1639–49. doi:10.1093/ije/dys210
40. Lind L, Elmståhl S, Bergman E, Englund M, Lindberg E, Michaelsson K, et al. EpiHealth: a large population-based cohort study for investigation of gene–lifestyle interactions in the pathogenesis of common diseases. Eur J Epidemiol. 2013 Feb 24;28(2):189–97. doi:10.1007/s10654-013-9787-x
41. Wittchen HU, Glaesmer H, März W, Stalla G, Lehnert H, Zeiher AM, et al. Cardiovascular risk factors in primary care: methods and baseline prevalence rates – the DETECT program. Curr Med Res Opin. 2005 Apr;21(4):619–29. doi:10.1185/030079905X38187
42. Orwoll E, Blank JB, Barrett-Connor E, Cauley J, Cummings S, Ensrud K, et al. Design and baseline characteristics of the osteoporotic fractures in men (MrOS) study — A large observational study of the determinants of fracture in older men. Contemp Clin Trials. 2005 Oct;26(5):569–85. doi:10.1016/j.cct.2005.05.006
43. Bai Y, Yang A, Pu H, Dai M, Cheng N, Ding J, et al. Cohort Profile: The China Metal-Exposed Workers Cohort Study (Jinchang Cohort). Int J Epidemiol. 2016 Nov 10;dyw223. doi:10.1093/ije/dyw223
44. Li L, Liu R, Jiang C, Du X, Huffman MD, Lam CSP, et al. Assessing the Evidence–Practice Gap for Heart Failure in China: The Heart Failure Registry of Patient Outcomes (HERO) Study Design and Baseline Characteristics. Eur J Heart Fail. 2020 Apr 1;22(4):646–60. doi:10.1002/ejhf.1630
45. Zhang Y, Chang Y, Ryu S, Cho J, Lee WY, Rhee EJ, et al. Thyroid hormone levels and incident chronic kidney disease in euthyroid individuals: the Kangbuk Samsung Health Study. Int J Epidemiol. 2014 Oct 1;43(5):1624–32. doi:10.1093/ije/dyu126
46. Kuno J, Tsubota-Utsugi M, Shimoda H, Takusari E, Takanashi N, Onoda T, et al. Association between changes in walking time and all-cause mortality among survivors of the Great East Japan Earthquake: the Research Project for Prospective Investigation of Health Problems Among Survivors of the Great East Japan Earthquake and Tsunami Disaster (RIAS) study. BMJ Public Health. 2025 Jan 9;3(1):e001321. doi:10.1136/bmjph-2024-001321
47. Van der Niepen P, Thijs V, Devroey D, Fieuw A, Dramaix M, Van de Borne P. Belgian global implementation of cardiovascular and stroke risk assessment study: methods and baseline data of the BELGICA-STROKE STUDY. European Journal of Cardiovascular Prevention & Rehabilitation. 2011 Aug 25;18(4):635–41. doi:10.1177/1741826710389416
48. Cohort Profile: The 45 and Up Study. Int J Epidemiol. 2008 Oct 1;37(5):941–7. doi:10.1093/ije/dym184
49. Peng H, Mao C, Zhang J, Wang F, Jiang H, Zhang Y, et al. Cohort Profile: Heart and Brain Investigation in Taicang (HABIT) study. Int J Epidemiol. 2024 Feb 1;53(1). doi:10.1093/ije/dyad192
50. Zhang Q, Huang Z, Xu T, Chen C, Xu Y, Yao D, et al. Health literacy and chronic disease prevalence: age-group differences in Zhejiang, China. Front Public Health. 2025 Sep 23;13. doi:10.3389/fpubh.2025.1602658
51. **Takeuchi K, Naito M, Kawai S, Tsukamoto M, Kadomatsu Y, Kubo Y, et al. Study Profile of the Japan Multi-institutional Collaborative Cohort (J-MICC) Study. J Epidemiol. 2021 Dec 5;31(12):JE20200147. doi:10.2188/jea.JE20200147**
52. Ghayour-Mobarhan M, Moohebati M, Esmaily H, Ebrahimi M, Parizadeh SMR, Heidari-Bakavoli AR, et al. Mashhad stroke and heart atherosclerotic disorder (MASHAD) study: design, baseline characteristics and 10-year cardiovascular risk estimation. Int J Public Health. 2015 Jul 6;60(5):561–72. doi:10.1007/s00038-015-0679-6
53. Teekaput C, Thiankhaw K, Wongcharoen W, Prasertwitayakij N, Gunaparn S, Phrommintikul A. Visit-to-visit lipid variability on long-term major adverse cardiovascular events: a prospective multicentre cohort from the CORE-Thailand registry. Sci Rep. 2025 Jan 14;15(1):1953. doi:10.1038/s41598-025-85453-w
54. Li M, Xu Y, Wan Q, Shen F, Xu M, Zhao Z, et al. Individual and Combined Associations of Modifiable Lifestyle and Metabolic Health Status With New-Onset Diabetes and Major Cardiovascular Events: The China Cardiometabolic Disease and Cancer Cohort (4C) Study. Diabetes Care. 2020 Aug 1;43(8):1929–36. doi:10.2337/dc20-0256
55. Peasey A, Bobak M, Kubinova R, Malyutina S, Pajak A, Tamosiunas A, et al. Determinants of cardiovascular disease and other non-communicable diseases in Central and Eastern Europe: Rationale and design of the HAPIEE study. BMC Public Health. 2006 Dec 18;6(1):255. doi:10.1186/1471-2458-6-255
56. van der Flier WM, Scheltens P. Amsterdam Dementia Cohort: Performing Research to Optimize Care. Journal of Alzheimer’s Disease. 2018 Mar 13;62(3):1091–111. doi:10.3233/JAD-170850
57. Religa D, Fereshtehnejad SM, Cermakova P, Edlund AK, Garcia-Ptacek S, Granqvist N, et al. SveDem, the Swedish Dementia Registry – A Tool for Improving the Quality of Diagnostics, Treatment and Care of Dementia Patients in Clinical Practice. PLoS One. 2015 Feb 19;10(2):e0116538. doi:10.1371/journal.pone.0116538
58. Feng L, Kong L, Dong X, Lai X, Zhang D, Ren B, et al. China Stroke Registry for Patients With Traditional Chinese Medicine (CASES-TCM): Rationale and Design of a Prospective, Multicenter, Observational Study. Front Pharmacol. 2021 Aug 31;12. doi:10.3389/fphar.2021.743883
59. Hispanic Community Health Study/Study of Latinos (HCHS/SOL). HCHS/SOL website. Available at: https://sites9.cscc.unc.edu/hchs/home (accessed 16 April 2026).
60. Vaara S, Nieminen MS, Lokki ML, Perola M, Pussinen PJ, Allonen J, et al. Cohort Profile: The Corogene study. Int J Epidemiol. 2012 Oct;41(5):1265–71. doi:10.1093/ije/dyr090
61. Bi Y, Lu J, Wang W, Mu Y, Zhao J, Liu C, et al. Cohort profile: risk evaluation of cancers in Chinese diabetic individuals: a longitudinal (REACTION) study. J Diabetes. 2014 Mar;6(2):147-57. doi: 10.1111/1753-0407.12108
62. The Charles Bronfman Institute for Personalized Medicine. BioMe® Biobank Program. Available at: <https://icahn.mssm.edu/research/ipm/programs/biome-biobank> (accessed 16 April 2026).
63. Al Kuwari H, Al Thani A, Al Marri A, Al Kaabi A, Abderrahim H, Afifi N, et al. The Qatar Biobank: background and methods. BMC Public Health. 2015 Dec 3;15(1):1208. doi:10.1186/s12889-015-2522-7
64. Westermann D, Goodman SG, Nicolau JC, Requena G, Maguire A, Chen JY, et al. Rationale and design of the long‐Term rIsk, clinical manaGement, and healthcare Resource utilization of stable coronary artery dISease in post–myocardial infarction patients (TIGRIS) study. Clin Cardiol. 2017 Dec 16;40(12):1197–204. doi:10.1002/clc.22837
65. Empana JP, Bean K, Guibout C, Thomas F, Bingham A, Pannier B, et al. Paris Prospective Study III: a study of novel heart rate parameters, baroreflex sensitivity and risk of sudden death. Eur J Epidemiol. 2011 Nov 1;26(11):887–92. doi:10.1007/s10654-011-9618-x
66. Lei J, Xu X, Ji Y, Yang Y, Yi Q, Chen H, et al. Rational and design of the China Pulmonary Thromboembolism Registry Study (CURES): A prospective multicenter registry. Int J Cardiol. 2020 Oct;316:242–8. doi:10.1016/j.ijcard.2020.05.087
67. Freedman VA, Kasper JD. Cohort Profile: The National Health and Aging Trends Study (NHATS). Int J Epidemiol. 2019 Aug 1;48(4):1044–1045g. doi:10.1093/ije/dyz109
68. Ni W, Peng X, Yuan X, Sun Y, Zhang H, Zhang Y, et al. Protocol for Shenzhen Ageing Cohort Study (SZ-ageing): a prospective observational cohort study of elderly disability and cognitive impairment. BMJ Open. 2023 Jan 18;13(1):e065761. doi:10.1136/bmjopen-2022-065761
69. Cao H, Li B, Peng W, Pan L, Cui Z, Zhao W, et al. Associations of long-term exposure to ambient air pollution with cardiac conduction abnormalities in Chinese adults: The CHCN-BTH cohort study. Environ Int. 2020;143:105981. doi: 10.1016/j.envint.2020.105981
70. Fuqua SR, Wyatt SB, Andrew ME, Sarpong DF, Henderson FR, Cunningham MF, Taylor HA Jr. Recruiting African-American research participation in the Jackson Heart Study: methods, response rates, and sample description. Ethn Dis. 2005 Autumn;15(4 Suppl 6):S6-18-29.
71. Chang WH, Sohn MK, Lee J, Kim DY, Lee SG, Shin YI, et al. Korean Stroke Cohort for functioning and rehabilitation (KOSCO): study rationale and protocol of a multi-centre prospective cohort study. BMC Neurol. 2015 Dec 25;15(1):42. doi:10.1186/s12883-015-0293-5
72. Penno G, Solini A, Zoppini G, Orsi E, Zerbini G, Trevisan R, et al. Rate and Determinants of Association Between Advanced Retinopathy and Chronic Kidney Disease in Patients With Type 2 Diabetes. Diabetes Care. 2012 Nov 1;35(11):2317–23. doi:10.2337/dc12-0628
73. Bergström G, Berglund G, Blomberg A, Brandberg J, Engström G, Engvall J, et al. The Swedish CArdioPulmonary BioImage Study: objectives and design. J Intern Med. 2015 Dec 19;278(6):645–59. doi:10.1111/joim.12384
74. Baber U, Mehran R, Sartori S, Schoos MM, Sillesen H, Muntendam P, et al. Prevalence, Impact, and Predictive Value of Detecting Subclinical Coronary and Carotid Atherosclerosis in Asymptomatic Adults. J Am Coll Cardiol. 2015 Mar;65(11):1065–74. doi:10.1016/j.jacc.2015.01.017
75. Poustchi H, Eghtesad S, Kamangar F, Etemadi A, Keshtkar AA, Hekmatdoost A, et al. Prospective Epidemiological Research Studies in Iran (the PERSIAN Cohort Study): Rationale, Objectives, and Design. Am J Epidemiol. 2018 Apr 1;187(4):647–55. doi:10.1093/aje/kwx314
76. Schram MT, Sep SJS, van der Kallen CJ, Dagnelie PC, Koster A, Schaper N, et al. The Maastricht Study: an extensive phenotyping study on determinants of type 2 diabetes, its complications and its comorbidities. Eur J Epidemiol. 2014 Jun 23;29(6):439–51. doi:10.1007/s10654-014-9889-0
77. Kim Y, Han BG. Cohort Profile: The Korean Genome and Epidemiology Study (KoGES) Consortium. Int J Epidemiol. 2017 Apr 1;46(2):e20–e20. doi:10.1093/ije/dyv316
78. Lahelma E, Aittomäki A, Laaksonen M, Lallukka T, Martikainen P, Piha K, et al. Cohort Profile: The Helsinki Health Study. Int J Epidemiol. 2013 Jun;42(3):722–30. doi:10.1093/ije/dys039
79. Kott KA, Vernon ST, Hansen T, Yu C, Bubb KJ, Coffey S, et al. Biobanking for discovery of novel cardiovascular biomarkers using imaging-quantified disease burden: protocol for the longitudinal, prospective, BioHEART-CT cohort study. BMJ Open. 2019 Sep 18;9(9):e028649. doi:10.1136/bmjopen-2018-028649
80. Mesnier J, Ducrocq G, Danchin N, Ferrari R, Ford I, Tardif JC, et al. International Observational Analysis of Evolution and Outcomes of Chronic Stable Angina: The Multinational CLARIFY Study. Circulation. 2021 Aug 17;144(7):512–23. doi:10.1161/CIRCULATIONAHA.121.054567
81. Laakso M, Kuusisto J, Stančáková A, Kuulasmaa T, Pajukanta P, Lusis AJ, et al. The Metabolic Syndrome in Men study: a resource for studies of metabolic and cardiovascular diseases. J Lipid Res. 2017 Mar;58(3):481–93. doi:10.1194/jlr.O072629
82. Bassil DT, Farrell MT, Wagner RG, Brickman AM, Glymour MM, Langa KM, et al. Cohort Profile Update: Cognition and dementia in the Health and Aging in Africa Longitudinal Study of an INDEPTH community in South Africa (HAALSI dementia). Int J Epidemiol. 2022 Aug 10;51(4):e217–26. doi:10.1093/ije/dyab250
83. Ko YA, Hayek S, Sandesara P, Samman Tahhan A, Quyyumi A. Cohort profile: the Emory Cardiovascular Biobank (EmCAB). BMJ Open. 2017 Dec 29;7(12):e018753. doi: 10.1136/bmjopen-2017-018753
84. Snijder MB, Galenkamp H, Prins M, Derks EM, Peters RJG, Zwinderman AH, et al. Cohort profile: the Healthy Life in an Urban Setting (HELIUS) study in Amsterdam, The Netherlands. BMJ Open. 2017 Dec;7(12):e017873. doi:10.1136/bmjopen-2017-017873
85. Engel C, Wirkner K, Zeynalova S, Baber R, Binder H, Ceglarek U, et al. Cohort Profile: The LIFE-Adult-Study. Int J Epidemiol. 2023 Feb 8;52(1):e66–79. doi:10.1093/ije/dyac114
86. Chen Z, Chen J, Collins R, Guo Y, Peto R, Wu F, et al. China Kadoorie Biobank of 0.5 million people: survey methods, baseline characteristics and long-term follow-up. Int J Epidemiol. 2011 Dec 1;40(6):1652–66. doi:10.1093/ije/dyr120
87. Wang F, Zhu J, Yao P, Li X, He M, Liu Y, et al. Cohort Profile: The Dongfeng–Tongji cohort study of retired workers. Int J Epidemiol. 2013 Jun;42(3):731–40. doi:10.1093/ije/dys053
88. Korb CA, Elbaz H, Schuster AK, Nickels S, Ponto KA, Schulz A, et al. Five-year cumulative incidence and progression of age-related macular degeneration: results from the German population-based Gutenberg Health Study (GHS). Graefes Arch Clin Exp Ophthalmol. 2022 Jan;260(1):55-64. doi: 10.1007/s00417-021-05312-y
89. Signorello LB, Hargreaves MK, Blot WJ. The Southern Community Cohort Study: investigating health disparities. J Health Care Poor Underserved. 2010 Feb;21(1 Suppl):26-37. doi: 10.1353/hpu.0.0245
90. Ahsan H, Chen Y, Parvez F, Argos M, Hussain AI, Momotaj H, et al. Health Effects of Arsenic Longitudinal Study (HEALS): Description of a multidisciplinary epidemiologic investigation. J Expo Sci Environ Epidemiol. 2006 Mar 1;16(2):191–205. doi:10.1038/sj.jea.7500449
91. Chin CT, Wang TY, Anstrom KJ, Zhu B, Maa JF, Messenger JC, et al. Treatment with Adenosine Diphosphate Receptor Inhibitors—Longitudinal Assessment of Treatment Patterns and Events after Acute Coronary Syndrome (TRANSLATE-ACS) study design: Expanding the paradigm of longitudinal observational research. Am Heart J. 2011 Nov;162(5):844–51. doi:10.1016/j.ahj.2011.08.021
92. Hercberg S, Castetbon K, Czernichow S, Malon A, Mejean C, Kesse E, et al. The Nutrinet-Santé Study: a web-based prospective study on the relationship between nutrition and health and determinants of dietary patterns and nutritional status. BMC Public Health. 2010 Dec 11;10(1):242. doi:10.1186/1471-2458-10-242
93. Avram R, Tison GH, Aschbacher K, Kuhar P, Vittinghoff E, Butzner M, et al. Real-world heart rate norms in the Health eHeart study. NPJ Digit Med. 2019 Jun 25;2(1):58. doi:10.1038/s41746-019-0134-9
94. Butler TL, Fraser GE, Beeson WL, Knutsen SF, Herring RP, Chan J, et al. Cohort Profile: The Adventist Health Study-2 (AHS-2). Int J Epidemiol. 2008 Apr;37(2):260–5. doi:10.1093/ije/dym165
95. Na L, Wu X, Feng R, Li J, Han T, Lin L, et al. The Harbin Cohort Study on Diet, Nutrition and Chronic Non-Communicable Diseases: Study Design and Baseline Characteristics. PLoS One. 2015 Apr 9;10(4):e0122598. doi:10.1371/journal.pone.0122598
96. Regan EA, Hokanson JE, Murphy JR, Make B, Lynch DA, Beaty TH, et al. Genetic Epidemiology of COPD (COPDGene) Study Design. COPD: Journal of Chronic Obstructive Pulmonary Disease. 2011 Feb 9;7(1):32–43. doi:10.3109/15412550903499522
97. Huang Y, Bi Y, Xu M, Ma Z, Xu Y, Wang T, et al. Nonalcoholic Fatty Liver Disease Is Associated With Atherosclerosis in Middle-Aged and Elderly Chinese. Arterioscler Thromb Vasc Biol. 2012 Sep;32(9):2321–6. doi:10.1161/ATVBAHA.112.252957
98. Kim JH, Chae SC, Oh DJ, Kim HS, Kim YJ, Ahn Y, et al. Multicenter Cohort Study of Acute Myocardial Infarction in Korea - Interim Analysis of the Korea Acute Myocardial Infarction Registry-National Institutes of Health Registry. Circ J. 2016 May 25;80(6):1427-36. doi: 10.1253/circj.CJ-16-0061
99. Song K, Du H, Zhang Q, Wang C, Guo Y, Wu H, et al. Serum Immunoglobulin M Concentration Is Positively Related to Metabolic Syndrome in an Adult Population: Tianjin Chronic Low-Grade Systemic Inflammation and Health (TCLSIH) Cohort Study. PLoS One. 2014 Feb 12;9(2):e88701. doi:10.1371/journal.pone.0088701
100. Guo Y, Yang J, Ma R, Zhang X, Guo H, He J, et al. Metabolic Dysfunction-Associated Fatty Liver Disease Is Associated with the Risk of Incident Cardiovascular Disease: A Prospective Cohort Study in Xinjiang. Nutrients. 2022 Jun 7;14(12):2361. doi:10.3390/nu14122361
101. Maggioni AP, Clark AL, Barrios V, Damy T, Drozdz J, Fonseca C, et al. Outcomes with Sacubitril/Valsartan in Outpatients with Heart Failure and Reduced Ejection Fraction: The ARIADNE Registry. ESC Heart Fail. 2022 Dec 1;9(6):4209–18. doi:10.1002/ehf2.14014
102. Baharudin N, Mohamed-Yassin MS, Daher AM, Ramli AS, Khan NAMN, Abdul-Razak S. Prevalence and factors associated with lipid-lowering medications use for primary and secondary prevention of cardiovascular diseases among Malaysians: the REDISCOVER study. BMC Public Health. 2022 Dec 4;22(1):228. doi:10.1186/s12889-022-12595-1
103. Battista S, Kiadaliri A, Jönsson T, Gustafsson K, Englund M, Testa M, et al. Factors Associated With Adherence to a Supervised Exercise Intervention for Osteoarthritis: Data From the Swedish Osteoarthritis Registry. Arthritis Care Res (Hoboken). 2023 Oct 12;75(10):2117–26. doi:10.1002/acr.25135
104. Li Z, Guo X, Zheng L, Sun Z, Yang H, Sun G, et al. Prehypertension in Rural Northeastern China: Results From the Northeast China Rural Cardiovascular Health Study. The Journal of Clinical Hypertension. 2014 Sep 6;16(9):664–70. doi:10.1111/jch.12378
105. Kurki MI, Karjalainen J, Palta P, Sipilä TP, Kristiansson K, Donner KM, et al. Author Correction: FinnGen provides genetic insights from a well-phenotyped isolated population. Nature. 2023 Mar 16;615(7952):E19–E19. doi:10.1038/s41586-023-05837-8
106. Kondal D, Patel SA, Ali MK, Mohan D, Rautela G, Gujral UP, et al. Cohort Profile: The Center for cArdiometabolic Risk Reduction in South Asia (CARRS). Int J Epidemiol. 2022 Dec 13;51(6):e358–71. doi:10.1093/ije/dyac014
107. Zhao P, Shi W, Shi Y, Xiong Y, Ding C, Song X, et al. Positive association between weight-adjusted-waist index and hyperuricemia in patients with hypertension: The China H-type hypertension registry study. Front Endocrinol (Lausanne). 2022 Oct 6;13:1007557. doi: 10.3389/fendo.2022.1007557
108. Hsieh CY, Wu DP, Sung SF. Registry-based stroke research in Taiwan: past and future. Epidemiol Health. 2018 Feb 4;40:e2018004. doi: 10.4178/epih.e2018004
109. Shimada H, Lee S, Doi T, Bae S, Tsutsumimoto K, Arai H. Prevalence of Psychological Frailty in Japan: NCGG-SGS as a Japanese National Cohort Study. J Clin Med. 2019 Sep 27;8(10):1554. doi:10.3390/jcm8101554
110. Xu H, Liu Q, Cao K, Ye Y, Zhang B, Li Z, et al. Distribution, Characteristics, and Management of Older Patients With Valvular Heart Disease in China. JACC: Asia. 2022 Jun;2(3):354–65. doi:10.1016/j.jacasi.2021.11.013
111. Augusto SN Jr, Wu Y, Chaikijurajai T, Hazen SL, Tang WHW. Abbreviated Duke Activity Status Index for Risk Stratification in Heart Failure. Am J Cardiol. 2025 Feb 15;237:54-59. doi: 10.1016/j.amjcard.2024.11.029
112. Sibarani MHR, Wijaya IP, Rizka A, Soewondo P, Riyadina W, Rahajeng E, et al. Cardiovascular disease prediction model for Indonesian adult population with prediabetes and diabetes mellitus: The Bogor Cohort study of Noncommunicable Diseases Risk Factors. Diabetes & Metabolic Syndrome: Clinical Research & Reviews. 2022 Jan;16(1):102330. doi:10.1016/j.dsx.2021.102330
113. Wang C, Song D, Dong J, Zhao Y, Liu Y, Gao J, et al. Dynamic Prediction of Cardiovascular Death among Old People with Mildly Reduced Kidney Function Using Deep Learning Models Based on a Prospective Cohort Study. Gerontology. 2025 Apr 3;71(6):474–87. doi:10.1159/000545679
114. Clark NG, Fox KM, Grandy S. Symptoms of Diabetes and Their Association With the Risk and Presence of Diabetes. Diabetes Care. 2007 Nov 1;30(11):2868–73. doi:10.2337/dc07-0816
115. Prior JA, Rushton CA, Jordan KP, Kadam UT. Comorbidity Cohort (2C) study: Cardiovascular disease severity and comorbid osteoarthritis in primary care. BMC Health Serv Res. 2012 Dec 3;12(1):295. doi:10.1186/1472-6963-12-295
116. Wyse DG. The Euro Heart Survey on atrial fibrillation: a picture and a thousand wordsThe opinions expressed in this article are not necessarily those of the Editors of the European Heart Journal or of the European Society of Cardiology. Eur Heart J. 2005 Nov 1;26(22):2356–7. doi:10.1093/eurheartj/ehi489
117. Heuzey JY Le, Breithardt G, Camm J, Crijns H, Dorian P, Kowey PR, et al. The RecordAF Study: Design, Baseline Data, and Profile of Patients According to Chosen Treatment Strategy for Atrial Fibrillation. Am J Cardiol. 2010 Mar;105(5):687–93. doi:10.1016/j.amjcard.2009.10.012
118. Heistaro S, ed. Methodology report: Health 2000 Survey. Helsinki: National Public Health Institute (KTL); 2008. Available at: https://urn.fi/URN:NBN:fi-fe201204193320 (Accessed 16 April 2026).
119. Harada S, Iida M, Miyagawa N, Hirata A, Kuwabara K, Matsumoto M, et al. Study Profile of the Tsuruoka Metabolomics Cohort Study (TMCS). J Epidemiol. 2024 Aug 5;34(8):JE20230192. doi:10.2188/jea.JE20230192
120. Casasnovas JA, Alcaide V, Civeira F, Guallar E, Ibañez B, Borreguero JJ, et al. Aragon workers’ health study – design and cohort description. BMC Cardiovasc Disord. 2012 Dec 19;12(1):45. doi:10.1186/1471-2261-12-45
121. Wang Y, Cui L, Ji X, Dong Q, Zeng J, Wang Y, et al. The China National Stroke Registry for Patients with Acute Cerebrovascular Events: Design, Rationale, and Baseline Patient Characteristics. International Journal of Stroke. 2011 Aug 17;6(4):355–61. doi:10.1111/j.1747-4949.2011.00584.x
122. Chen YJ, Lin CF, Feng J, Chiu HL. Influencing factors of participation in and satisfaction with elderly health checkups: a cross-sectional study. Front Public Health. 2023 Apr 27;11. doi:10.3389/fpubh.2023.1104438
123. Bueno H, Danchin N, Tafalla M, Bernaud C, Annemans L, Van de Werf F. EPICOR (long-tErm follow-up of antithrombotic management Patterns In acute CORonary syndrome patients) study: rationale, design, and baseline characteristics. Am Heart J. 2013 Jan;165(1):8–14. doi:10.1016/j.ahj.2012.10.018
124. Zhao Y, Sun H, Wang B, Zhang M, Luo X, Ren Y, et al. Impaired fasting glucose predicts the development of hypertension over 6 years in female adults: Results from the rural Chinese cohort study. J Diabetes Complications. 2017 Jul;31(7):1090–5. doi:10.1016/j.jdiacomp.2017.04.006
125. Lee MY, Kim MY, Kim SY, Kim JH, Kim BH, Shin JY, et al. Association between alcohol intake amount and prevalence of metabolic syndrome in Korean rural male population. Diabetes Res Clin Pract. 2010 May;88(2):196–202. doi:10.1016/j.diabres.2010.01.009
126. Lupilov A, Krause D, Klaassen-Mielke R, Trampisch HJ, Rudolf H. Effects of Three Different Methods Defining Onset of Peripheral Artery Disease on the Assessments of Incidence and Important Predictors - Results from the German Epidemiological Trial on Ankle Brachial Index (getABI). Vasc Health Risk Manag. 2021 Jul 24;17:421-429. doi: 10.2147/VHRM.S307675
127. Yamada Y, Matsui K, Takeuchi I, Oguri M, Fujimaki T. Association of genetic variants with hypertension in a longitudinal population-based genetic epidemiological study. Int J Mol Med. 2015 May;35(5):1189–98. doi:10.3892/ijmm.2015.2151
128. Reyes‐Dumeyer D, Faber K, Vardarajan B, Goate A, Renton A, Chao M, et al. The National Institute on Aging Late‐Onset Alzheimer’s Disease Family Based Study: A resource for genetic discovery. Alzheimer’s & Dementia. 2022 Oct 3;18(10):1889–97. doi:10.1002/alz.12514
129. Setoh K, Matsuda F. Cohort Profile: The Nagahama Prospective Genome Cohort for Comprehensive Human Bioscience (The Nagahama Study). In. 2022. p. 127–43. doi:10.1007/978-981-16-5727-6_7
130. Hadamitzky M, Achenbach S, Al-Mallah M, Berman D, Budoff M, Cademartiri F, et al. Optimized Prognostic Score for Coronary Computed Tomographic Angiography. J Am Coll Cardiol. 2013 Jul;62(5):468–76. doi:10.1016/j.jacc.2013.04.064
131. Cowie MR, Komajda M. Quality of Physician Adherence to Guideline Recommendations for Life-saving Treatment in Heart Failure: an International Survey. Card Fail Rev. 2017;3(2):130. doi:10.15420/cfr.2017:13:1
132. Zheng R, Yang T, Chen Q, Chen C, Mao Y. Serum Uric Acid Concentrations Can Predict Hypertension: A Longitudinal Population-Based Epidemiological Study. Hormone and Metabolic Research. 2017 Nov 18;49(11):873–9. doi:10.1055/s-0043-119129
133. Hsu CC, Chang HY, Wu IC, Chen CC, Tsai HJ, Chiu YF, et al. Cohort Profile: The Healthy Aging Longitudinal Study in Taiwan (HALST). Int J Epidemiol. 2017 Aug 1;46(4):1106–1106j. doi:10.1093/ije/dyw331
134. The “All of Us” Research Program. New England Journal of Medicine. 2019 Aug 15;381(7):668–76. doi:10.1056/NEJMsr1809937
135. Sääksjärvi K, Elonheimo HM, Ikonen J, Lehtoranta L, Parikka S, Härkänen T, et al. Cohort Profile: Healthy Finland Survey. Int J Epidemiol. 2024 Dec 16;54(1). doi:10.1093/ije/dyae166
136. Ferreccio C, Huidobro A, Cortés S, Bambs C, Toro P, Van De Wyngard V, et al. Cohort Profile: The Maule Cohort (MAUCO). Int J Epidemiol. 2020 Jun 1;49(3):760–761i. doi:10.1093/ije/dyaa003
137. Wang X, Mina T, Sadhu N, Jain PR, Ng HK, Low DY, et al. The Health for Life in Singapore (HELIOS) Study: delivering precision medicine research for Asian populations. Nat Commun. 2025 Dec 30;17(1):1. doi:10.1038/s41467-025-65774-0
138. Shafiq M, Fong AYY, Tai ES, Nang EEK, Wee HL, Adam J, et al. Cohort Profile: LIFE course study in CARdiovascular disease Epidemiology (LIFECARE). Int J Epidemiol. 2018 Oct 1;47(5):1399–1400g. doi:10.1093/ije/dyy168
139. Olson JE, Ryu E, Johnson KJ, Koenig BA, Maschke KJ, Morrisette JA, et al. The Mayo Clinic Biobank: A Building Block for Individualized Medicine. Mayo Clin Proc. 2013 Sep;88(9):952–62. doi:10.1016/j.mayocp.2013.06.006
140. Jamal R, Syed Zakaria SZ, Kamaruddin MA, Abd Jalal N, Ismail N, Mohd Kamil N, et al. Cohort Profile: The Malaysian Cohort (TMC) project: a prospective study of non-communicable diseases in a multi-ethnic population. Int J Epidemiol. 2015 Apr 1;44(2):423–31. doi:10.1093/ije/dyu089
141. Dhalla A, McDonald TE, Gallagher RP, Spinelli JJ, Brooks-Wilson AR, Lee TK, et al. Cohort Profile: The British Columbia Generations Project (BCGP). Int J Epidemiol. 2019 Apr 1;48(2):377–378k. doi:10.1093/ije/dyy160
142. Liu X, Mao Z, Li Y, Wu W, Zhang X, Huo W, et al. Cohort Profile: The Henan Rural Cohort: a prospective study of chronic non-communicable diseases. Int J Epidemiol. 2019 Dec 1;48(6):1756–1756j. doi:10.1093/ije/dyz039
143. Kirsh VA, Skead K, McDonald K, Kreiger N, Little J, Menard K, et al. Cohort Profile: The Ontario Health Study (OHS). Int J Epidemiol. 2023 Apr 19;52(2):e137–51. doi:10.1093/ije/dyac156
144. Cook MB, Sanderson SC, Deanfield JE, Reddington F, Roddam A, Hunter DJ, et al. Our Future Health: a unique global resource for discovery and translational research. Nat Med. 2025 Mar;31(3):728-730. doi: 10.1038/s41591-024-03438-0
145. Prasad K, Dwivedi SN, Kant S, Vibha D, Pandit AK, Karthikeyan G, et al. Cohort Profile: The LoCARPoN—a population-based prospective cohort study in middle-aged and older adults in India. Int J Epidemiol. 2022 Feb 18;51(1):29–30m. doi:10.1093/ije/dyab078
146. van der Sluijs KM, Bakker EA, Schoofs MCA, Vloet JIA, Verbeek ALM, Hopman MTE, et al. Cohort Profile: The Nijmegen Exercise Study (NES). Int J Epidemiol. 2025 Jun 11;54(4). doi:10.1093/ije/dyaf092
147. Chiba I, Nakaya N, Kogure M, Hatanaka R, Nakaya K, Tokioka S, et al. Associations between housing and psychological damage by earthquake and modifiable risk factors for dementia in general older adults: Tohoku Medical Megabank community‐based cohort study. Geriatr Gerontol Int. 2024 Jun 3;24(6):509–16. doi:10.1111/ggi.14867
